# Inner mitochondrial membrane protein Prohibitin 1 mediates Nix-induced, Parkin-independent mitophagy

**DOI:** 10.1038/s41598-022-26775-x

**Published:** 2023-01-02

**Authors:** Kibrom M. Alula, Yaritza Delgado-Deida, Rosemary Callahan, Andreas Till, Lucia Underwood, Winston E. Thompson, Rhonda F. Souza, Themistocles Dassopoulos, Joseph Onyiah, K. Venuprasad, Arianne L. Theiss

**Affiliations:** 1 Division of Gastroenterology and Hepatology, Department of Medicine, University of Colorado School of Medicine, 12700 East 19th Avenue, RC2 Campus Box: B158 HSC, Aurora, CO USA; 2 Department of Internal Medicine, University Hospital Bonn, Bonn, Germany; 3 Department of Obstetrics and Gynecology, Morehouse School of Medicine, Atlanta, GA USA; 4 Center for Esophageal Diseases, Baylor University Medical Center and Center for Esophageal Research, Baylor Scott & White Research Institute, Dallas, TX USA; 5 College of Medicine, University of Texas Southwestern Medical Center, Dallas, TX USA

**Keywords:** Mitochondria, Small intestine, Autophagy

## Abstract

Autophagy of damaged mitochondria, called mitophagy, is an important organelle quality control process involved in the pathogenesis of inflammation, cancer, aging, and age-associated diseases. Many of these disorders are associated with altered expression of the inner mitochondrial membrane (IMM) protein Prohibitin 1. The mechanisms whereby dysfunction occurring internally at the IMM and matrix activate events at the outer mitochondrial membrane (OMM) to induce mitophagy are not fully elucidated. Using the gastrointestinal epithelium as a model system highly susceptible to autophagy inhibition, we reveal a specific role of Prohibitin-induced mitophagy in maintaining intestinal homeostasis. We demonstrate that Prohibitin 1 induces mitophagy in response to increased mitochondrial reactive oxygen species (ROS) through binding to mitophagy receptor Nix/Bnip3L and independently of Parkin. Prohibitin 1 is required for ROS-induced Nix localization to mitochondria and maintaining homeostasis of epithelial cells highly susceptible to mitochondrial dysfunction.

## Introduction

Mitochondria are dynamic organelles that serve numerous cellular functions including energy production via oxidative metabolism, apoptosis induction, reactive oxygen species (ROS) production, immune signaling, and calcium storage and regulation. The cell possesses multiple quality control mechanisms to maintain the health of the mitochondrial population. This includes the removal of damaged mitochondria through mitophagy, a selective form of autophagy that targets mitochondria^1^. Autophagy is an evolutionarily conserved catabolic pathway that removes cytoplasmic components through lysosomal degradation, thereby promoting cell survival in response to stressors such as starvation, damaged organelles (mitochondria, endoplasmic reticulum, lysosomes, peroxisomes, endosomes), or the invasion of microorganisms^2^. Autophagy deficiency via genetic deletion demonstrates its importance in cell and tissue homeostasis, with the intestine being highly susceptible to autophagy inhibition in both epithelial and innate immune cells^3,4^. In fact, polymorphism in the autophagy gene *ATG16L1* confers susceptibility to Crohn’s disease, an inflammatory bowel disease^5^. In regards to the intestinal epithelium, autophagy is especially important in terminally differentiated long-lived cells, such as secretory Paneth cells in the crypt base in which damaged organelles are not diluted by cell replication^6^. Additionally, autophagy is crucial for intestinal secretory cell (Paneth and goblet cell) function^7,8^. Mice deficient in autophagy genes *Atg16l1*, *Atg7*, or *Atg5* manifest robust Paneth cell defects associated with the accumulation of damaged mitochondria^9–11^. However, a limitation of these autophagy-deficient models is the inability to discern the specific impact of mitophagy in the resulting pathology.

During autophagy activation, the cytosolic form of LC3 (LC3I) is proteolytically processed and conjugated to phosphatidylethanolamine to form lipidated LC3II which is recruited to autophagosomal membranes. Autophagosomes fuse with lysosomes to form so-called autolysosomes in which parts of the former autophagosomes and their cargo are degraded. The incorporation of damaged or unwanted mitochondria into autophagosomes is facilitated by the binding of mitochondrial membrane-localized autophagy receptors to LC3II in the forming phagosome via their LC3-interacting region (LIR)^12^. Some autophagy receptors are dependent on PINK1-mediated activation of the E3 ubiquitin ligase Parkin, which ubiquitinates outer mitochondrial membrane (OMM) proteins. Polyubiquitinated chains are then recognized by the autophagy receptor, thereby incorporating the mitochondrion into the autophagy pathway for degradation. Parkin-dependent autophagy receptors that have been identified to target mitochondria (and other polyubiquitinated targets) include p62, NBR1, Optineurin, and NDP52^13,14^. Other autophagy receptors act independently of Parkin and upon expression constitutively localize to the OMM. Parkin-independent autophagy receptors shown to target mitochondria include Bnip3, Nix/Bnip3L, FUNDC1, and BCl2L13^15^.

Much research has focused on events at the OMM involved in mitophagy. However, mitophagy is activated by mitochondrial dysfunction (depolarization, excessive misfolded proteins, accumulation of mitochondrial ROS, aberrant paternal transmission of mitochondrial DNA) occurring at the inner mitochondrial membrane (IMM) and matrix^16,17^. The mechanism(s) whereby this internal dysfunction stimulates events at the OMM to induce mitophagy are not completely elucidated. Given that severe mitochondrial dysfunction induces OMM rupture that is accompanied by the release of mitochondrial apoptotic proteins into the cytoplasm such as Cytochrome C^18^, we hypothesized that IMM proteins serve as detectors of internal mitochondrial dysfunction prior to OMM rupture to activate mitophagy and limit mitochondrial-induced apoptosis.

Prohibitin (PHB) 1 belongs to a family of proteins that share an evolutionarily conserved stomatin/prohibitin/flotillin/HflK/C (SPFH) domain, is ubiquitously expressed, and is highly abundant in the IMM^19^. A large C-terminal proportion of PHB1 (amino acids 28–272) reaches into the intermembrane mitochondrial space^19–21^, facilitating protein–protein interactions including interaction with OMM-localized proteins such as Miro1 and Miro2^22^. Known mitochondrial functions of PHB1 include stabilizing mitochondrial DNA-encoded proteins, regulating IMM fusion, and interacting with and maintaining optimal activity of the electron transport chain (ETC)^20,23,24^. Alteration of PHB1 expression is associated with various diseases including cancer, inflammatory bowel diseases, obesity, cholestatic liver diseases, and neurodegenerative diseases including Parkinson’s and Alzheimer’s disease^25–27^. In regards to mitophagy, a recent study revealed that PHB2, but not PHB1, acts as a Parkin-dependent mitophagy receptor during OMM rupture by direct binding to LC3 via its LIR domain^28^. We recently demonstrated that mice with gastrointestinal epithelial cell (IEC)-specific deletion of *Phb1* (*Phb1*^*iΔ*IEC^) manifest spontaneous Crohn’s disease-like intestinal inflammation driven by mitochondrial dysfunction and Paneth cell defects^29^. This ileitis is characterized by mucosal CD4^+^ T cell infiltration, villus blunting, crypt elongation, thickening of the muscularis layers, and gut microbial dysbiosis^29^. Here, using these mice with inducible deletion of *Phb1* in the gastrointestinal epithelium, a model system highly susceptible to autophagy inhibition, we reveal a concomitant deficiency in PHB2 and a role of mitophagy inhibition associated with the development of Crohn’s disease-like ileitis in this model.

## Results

### Mitophagy is inhibited during loss of PHB1

**Figure 1:**
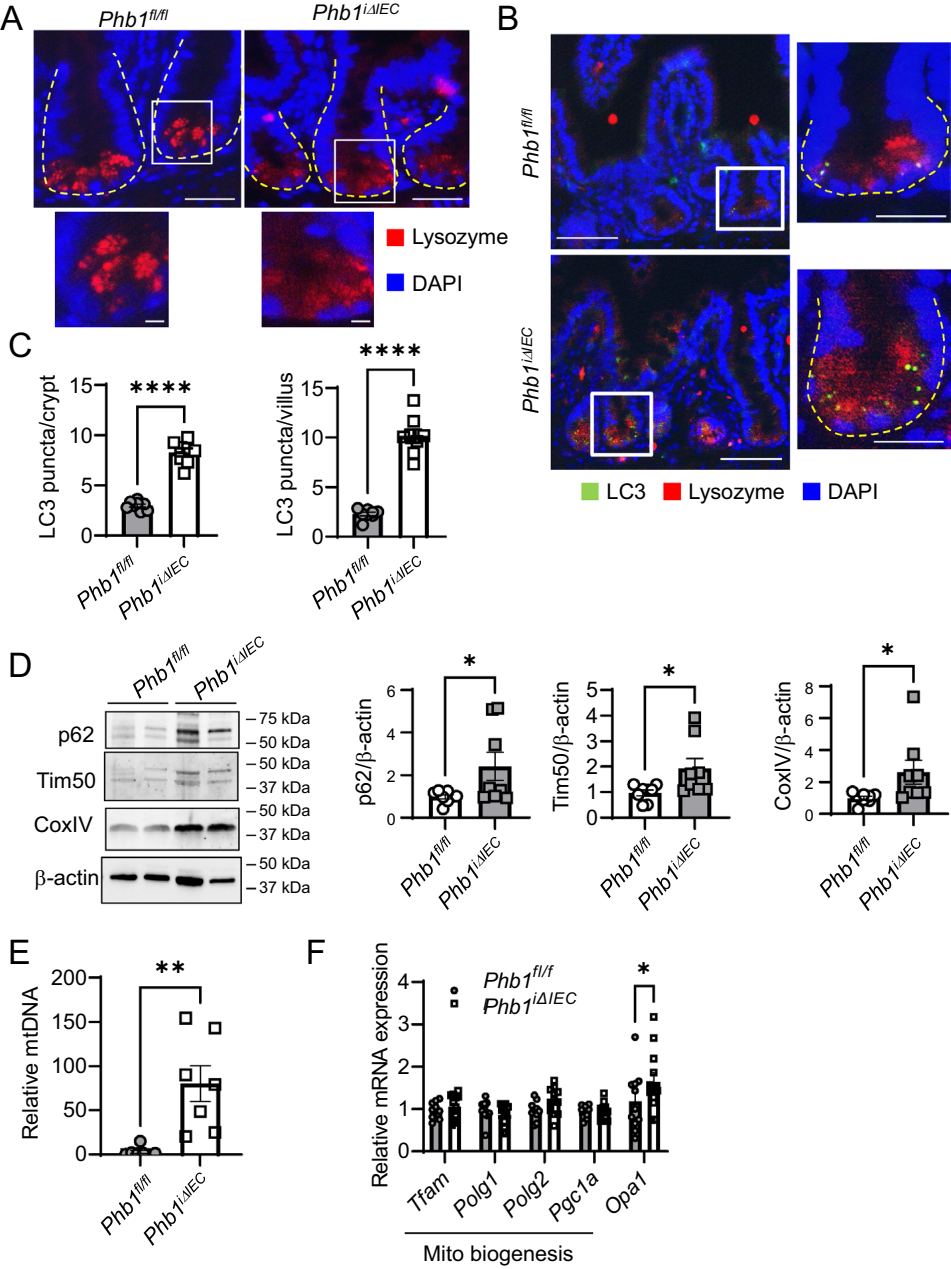
*Phb1*^*iΔIEC*^ mice exhibit increased autophagosome formation and decreased mitophagy in IECs. (**A**) Immunofluorescent (IF) lysozyme staining of ileum 1 week after induction of *Phb1* deletion. Dashed line marks crypt base. Scale bars: 50 μm, boxed pullout: 10 μm. (**B**) LC3II puncta in Paneth cells marked by lysozyme^+^ staining. Scale bars: 100 μm, boxed pullout: 50 μm. (**C**) The number of LC3II puncta across 50 crypts or 50 villi per mouse. (**D**) p62, Tim50, and CoxIV expression in isolated ileal IECs by western blot. Each lane represents one individual animal. (**E**) Relative mitochondrial/nuclear DNA level in isolated ileal IECs. (**F**) Mitochondrial biogenesis markers in isolated ileal IECs by qRT-PCR analysis. Results are presented as individual data points ± SEM of 7 mice (**A**,**E**), 8 mice (**D**), or 10–12 mice (**F**) each genotype *P < 0.05, **P < 0.01, ****P < 0.001 by unpaired, 2-tailed Student’s t test. See also Fig. S2 for full-length blots.

*Phb1*^*iΔIEC*^ mice exhibit loss of intestinal epithelial PHB1 expression (Fig. S1A) and manifest spontaneous ileal inflammation driven by mitochondrial dysfunction in IECs within 12 weeks after the induction of *Phb1* deletion^29^. Paneth cell defects, such as altered lysozyme granule staining (diffuse, disordered, and depleted), decreased electron-dense secretory granules, and vesiculated ER, were evident prior to inflammation as early as 1 week after the induction of *Phb1* deletion (Figs. 1A and S1B)^29^. Since these Paneth cell defects are reminiscent of mice deficient in autophagy genes *Atg16l1*, *Atg7*, or *Atg5*^9^, we measured autophagosome formation by LC3II in ileal IECs of *Phb1*^*fl/fl*^ and *Phb1*^*iΔIEC*^ mice. One week after the induction of *Phb1* deletion, autophagosomes were visualized as LC3II^+^ puncta and were increased in the crypt base in Paneth cells marked by lysozyme^+^ staining and in villi in secretory goblet cells marked by Muc2^+^ staining (Figs. 1B,C and S1C). This same increase in LC3II puncta has been demonstrated previously in Paneth and goblet cells of autophagy deficient mice^7,8^. Of note, goblet cells of *Phb1*^*iΔIEC*^ mice also exhibit abnormalities during the progression to ileitis, but are less susceptible to mitochondrial dysfunction than Paneth cells^29^. Increased autophagosomes during *Phb1* deletion could result from increased autophagy activation or decreased autophagosome degradation. Since abundance of p62 is a well-established marker of autophagosome turnover, we next assayed p62 in freshly isolated ileal IECs of *Phb1*^*iΔIEC*^ mice. p62 protein expression was variable across ileal IECs of *Phb1*^*iΔIEC*^ mice with some demonstrating increased abundance (Figs. 1D and S2), suggesting inhibition of autophagosome turnover in these *Phb1*^*iΔIEC*^ samples. As a measure of mitochondrial turnover, abundance of inner mitochondrial membrane proteins Tim50 and CoxIV was increased in IECs of some *Phb1*^*iΔIEC*^ mice (Figs. 1D and S2). As a more sensitive measure of mitochondrial turnover, mitochondrial DNA:nuclear DNA ratio was increased in IECs of *Phb1*^*iΔIEC*^ mice compared to *Phb1*^*fl/fl*^ mice (Fig. 1E). These increases in mitochondrial markers were not associated with increased mitochondrial biogenesis in *Phb1*^*iΔIEC*^ ileal IECs (Fig. 1F), as compared to *Opa1* expression, a regulator of mitochondrial IMM fusion that has been previously shown to be regulated by PHB1 expression^30^.

**Figure 2:**
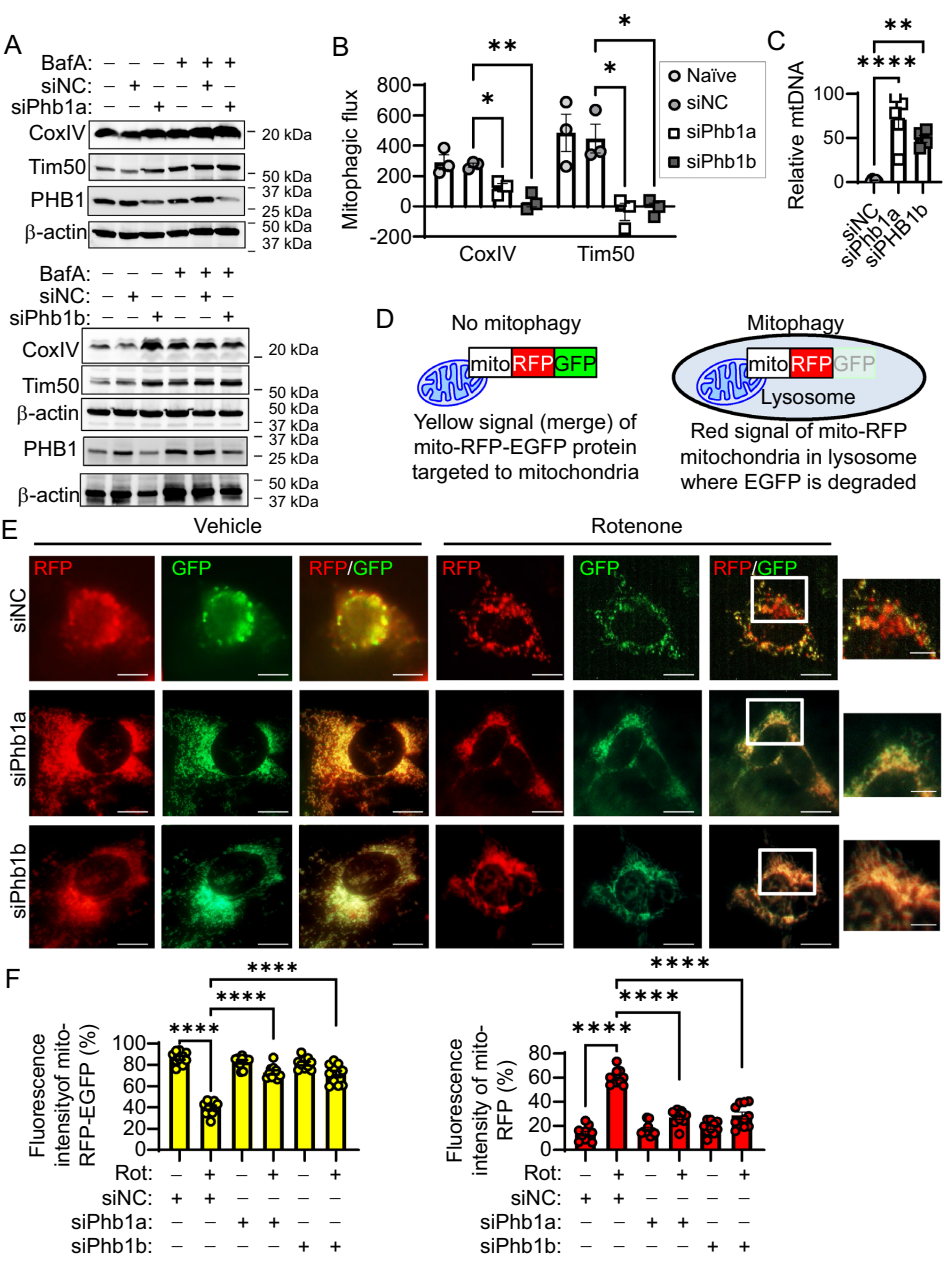
Mitochondria are not efficiently degraded during PHB1 deficiency. Mode-K intestinal epithelial cells were transfected with two independent siPhb1 constructs (designated as a and b) or siNegative Control construct (siNC). (**A**) Cells were treated with 10 nM Bafilomycin A1 (BafA) for 1 h and markers of mitophagy (CoxIV, Tim50) were measured by western blot. (**B**) Mitophagic flux. (**C**) Relative mitochondrial/nuclear DNA level in Mode-K cells. (**D**) Cartoon depiction of mito-RFP-EGFP indicator of mitophagy. (**E**,**F**) Mode-K cells were co-transfected with mito-RFP-EGFP during siPhb1 knockdown and treated with 500 nM rotenone for 2 h to induce mitophagy. (**E**) IF staining of mito-RFP-EGFP. Scale bars: 10 μm, boxed pullout: 5 μm. (**F**) Quantitation of yellow and red pixel intensity using the average of 50 cells per well across 10 wells per treatment. Results are presented as individual data points ± SEM of 1 per treatment group performed in 3 independent experiments (**A**,**B**) or 5 per treatment group (**C**). *P < 0.05, **P < 0.01, ****P < 0.001 vs. siNC veh by one-way ANOVA and Tukey’s posthoc test. See also Figs. S3 and S4 for full-length blots.

These results were corroborated in vitro using the non-transformed murine intestinal epithelial cell line, Mode-K, transfected with 2 independent siRNA constructs targeting PHB1 and treated with Bafilomycin A1 (BafA) to prevent autophagosome degradation. PHB1 knockdown induced accumulation of CoxIV and Tim50 similar to that induced by BafA alone (Figs. 2A, S3, S4). Compared to siNC (siRNA negative control) cells treated with BafA, CoxIV and Tim50 failed to further increase in siPHB1 cells treated with BafA (Figs. 2A, S3, S4), indicating decreased mitophagic flux during PHB1 knockdown (Fig. 2B). Furthermore, relative mitochondrial DNA:nuclear DNA ratio was increased in siPHB1 cells (Fig. 2C).

We next utilized expression of a mitochondrial-targeted fluorescent tag, mito-RFP-EGFP, to measure activation of mitophagy in Mode-K cells. Using this tag, intact mitochondria are marked by RFP-EGFP merged yellow signal under basal conditions, whereas during activation of mitophagy, mitochondria appear RFP^+^ since EGFP is quenched and finally degraded in the lysosome (Fig. 2D)^31^. Optimal conditions to elicit mitochondrial stress prior to the induction of apoptosis or severe mitochondrial alterations such as OMM rupture was determined in Mode-K cells treated with the ETC inhibitors rotenone, oligomycin, and antimycin A from 2 to 16 h (Figs. S5 and S6). Rotenone, oligomycin, and antimycin A increased the formation of mitochondrial ROS after 2 h of treatment at a similar magnitude (Fig. S5A). Unlike antimycin A, rotenone did not stimulate cytochrome C release from mitochondria to the cytosol at 2 or 8 h of treatment (Fig. S5B,C). Additionally, only 31% of mitochondria exhibited OMM rupture after 16 h of rotenone treatment, which was significantly less severe than oligomycin or antimycin A (Fig. S6B), as previously reported^28,32^. Therefore, rotenone treatment for 2 h was utilized to induce mitochondrial stress prior to the induction of apoptosis or OMM rupture. In Mode-K cells transfected with siNC, treatment with rotenone for 2 h activated mitophagy as indicated by increased mito-RFP-EGFP-induced red signal and decreased yellow signal (Figs. 2E,F, and S7). On the contrary, cells deficient in PHB1 showed little to no activation of mitophagy by rotenone (Figs. 2E,F, and S7). Collectively, these results suggest that mitochondria are not efficiently degraded during PHB1 deficiency in non-transformed IECs.

### PHB1 serves as an inducer of mitophagy during increased mitochondrial ROS independently of PHB2

**Figure 3:**
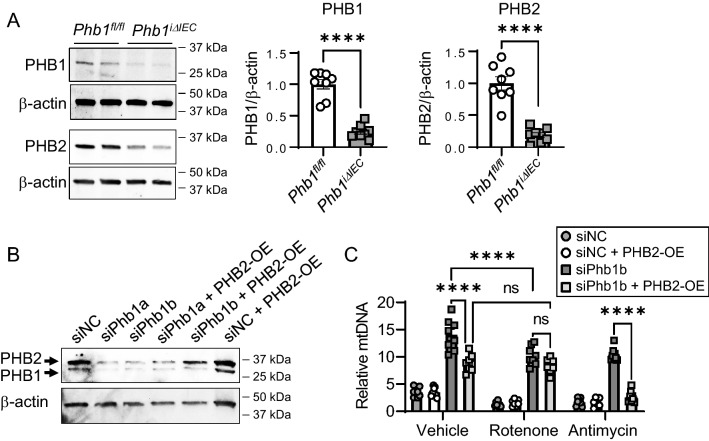
Restored expression of PHB2 during loss of PHB1 restores mitophagy induced by antimycin A but not rotenone. (**A**) Western blots of protein from isolated ileal IECs. Each lane represents one individual animal. (**B**) Efficiency of PHB1 knockdown and PHB2 overexpression in Mode-K cells were measured by western blot. (**C**) Mode-K cells were co-transfected with siPHB1b or siNC constructs and PHB2 overexpression plasmid (PHB2-OE) or empty vector control and treated with 500 nM rotenone or 100 nM antimycin A for 2 h. Relative mitochondrial/nuclear DNA level in Mode-K cells as indicator of mitophagy. Results are presented as individual data points ± SEM of 8 mice per group (**A**) or 8 per treatment group (**C**). ****P < 0.001 by unpaired, 2-tailed Student’s t test (**A**) or two-way ANOVA and Tukey’s posthoc test (**C**). See also Figs. S8, S9, and S10 for full-length blots.

PHB1 and PHB2 have both independent and dependent functions that are at least, in part, regulated by cellular localization and numerous post-translational modifications^33^. For instance, previous studies demonstrated a role of PHB2 independent of PHB1 in mitophagy activation during outer mitochondrial membrane rupture through direct binding to LC3II^28^. We therefore tested whether inhibited mitophagy during PHB1 deletion in IECs was associated with a compensatory loss in PHB2 expression. Indeed, freshly isolated ileal IECs from *Phb1*^*iΔIEC*^ mice exhibited a concomitant loss of PHB1 and PHB2 expression (Figs. 3A, S8, and S9) with similar results in Mode-K cells during siPhb1 knockdown (Figs. 3B and S10). This linked expression of PHB1 and PHB2 has been shown previously in other systems^20,28^. Forced overexpression (OE) of PHB2 was able to restore PHB2 levels during siPhb1b knockdown (Qiagen construct) but not during siPhb1a knockdown (Invitrogen construct) (Fig. 3B), therefore siPhb1b knockdown coupled with PHB2 exogenous expression was used for further analysis. Forced expression of PHB2 lessened the inhibition of basal mitophagy by siPhb1b in vehicle-treated cells as indicated by decreased relative mtDNA accumulation (Fig. 3C). This suggests concomitant loss of PHB2 (as shown in Fig. 3A,B) is partially responsible for basal mitophagy inhibition during PHB1 knockdown. Rotenone treatment for 2 h induces mitophagy in siPhb1b cells with a slight but significant decrease in relative mtDNA accumulation in rotenone-treated siPhb1b cells compared to vehicle-treated siPhb1b cells (Fig. 3C). This is likely due to remaining PHB1 and PHB2 expression since the siPhb1b construct produces incomplete knockdown of PHB1 and incomplete compensatory loss of PHB2 (as shown in Fig. 3B). Interestingly, forced overexpression of PHB2 had no effect on relative mtDNA accumulation (Fig. 3C) or mito-RFP-EGFP expression (Fig. S11) during siPhb1b knockdown and rotenone treatment, suggesting PHB2 cannot rescue mitophagy response to rotenone during PHB1 deficiency. We reasoned this could be due to PHB2 requiring OMM rupture to induce mitophagy activation^28^ coupled with 2 h rotenone not stimulating OMM rupture in our system (Figs. S5 and S6). We therefore treated with antimycin A to induce OMM rupture (Figs. S5B and S6) and noted that forced expression of PHB2 rescued antimycin A-induced relative mtDNA accumulation (Fig. 3C) or mito-RFP-EGFP expression (Fig. S11) during siPhb1b knockdown. These results suggest that mitophagy induction without robust OMM rupture (rotenone treatment) requires PHB1 independently of PHB2.

### PHB1 interacts with mitophagy receptor Nix independently of Parkin

**Figure 4:**
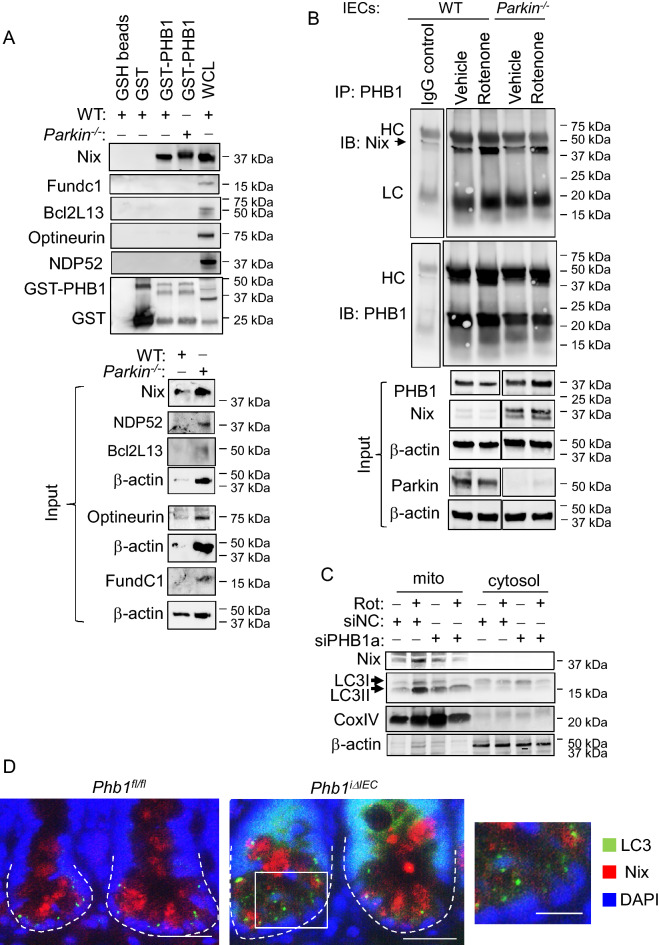
PHB1 interacts with the mitophagy receptor Nix independently of Parkin and is necessary for rotenone-induced Nix accumulation in mitochondria. (**A**) Protein from isolated ileal IECs of wild-type (WT) or *Parkin*^*−/−*^ mice was incubated with GST-PHB1 and analyzed by western blotting. Protein exposed to GSH beads or GST alone were used as controls. Whole-cell lysate was included on western blots to ensure antibody signal. (**B**) Primary ileum IECs isolated from WT or *Parkin*^*−/−*^ mice were treated with 500 nM rotenone or vehicle for 2 h. Protein was immunoprecipitated (IP) using a PHB1 antibody or control IgG antibody and immunoblotted (IB) for Nix or PHB1. HC: heavy chain, LC: light chain. Images are cropped from the same blots. (**C**) Mode-K cells were treated with 500 nM rotenone for 2 h and mitochondrial and cytosolic extracts were isolated for western blotting. CoxIV (mitochondrial marker) and β-actin (cytosolic marker) were used to denote purity of subcellular isolation. (**D**) IF staining of ileal crypts. Dashed line denotes crypt base. Scale bars: 50 μm, boxed pullout: 25 μm. See also Figs. S14–S18 for full-length blots.

Previous studies have established that Parkin-dependent mitophagy requires rupture of the OMM^28,34^. However, Parkin-independent mitophagy does not require OMM rupture; Parkin-independent autophagy receptors localize to the OMM to mediate mitophagy. In fact, Nix-mediated mitophagy has been shown to be induced by mitochondrial ROS accumulation without rupture of the OMM^35^. We next determined whether PHB1 interacts with mitophagy receptors and whether Parkin was involved. The mitophagy receptor Nix interacted with GST-PHB1 in protein isolated from ileal IECs of wild-type (WT) or *Parkin*^*−/−*^ mice and Mode-K cells (Figs. 4A, S12A, S14, and S15). There was no interaction between GST-PHB1 and other mitophagy receptors including FundC1, Bnip3, Bcl2L13, Optineurin, or NDP52 (Fig. 4A). Parkin independent interaction of PHB1 with Nix was confirmed by co-immunoprecipitation assay (Figs. 4B, S16, S17). Additionally, PHB1/Nix interaction was increased after 2 h rotenone treatment (Fig. 4B). Induction of PHB1 interaction with Nix during mitochondrial stress induced by rotenone is conserved in cell types beyond IECs including HeLa cells (Fig. S12B).

Induction of mitophagy by Nix involves its transcriptional upregulation, constitutive localization to the OMM, and direct interaction with LC3II^15^. During siPHB1 knockdown in Mode-K cells, there was ample rotenone-induced Nix transcriptional activation (Fig. S12C) but Nix did not accumulate in mitochondria during rotenone treatment (Figs. 4C and S17). Rotenone-induced LC3II association with the mitochondrial fraction was inhibited during siPHB1 knockdown (Fig. 4C). Although *Phb1*^*iΔIEC*^ mice exhibited similar Nix expression in ileal IECs as *Phb1*^*fl/fl*^ mice (Fig. S12D), less Nix interacted with LC3II in crypts of *Phb1*^*iΔIEC*^ mice, despite high expression of LC3II (Fig. 4D). Together, these results suggest that the IMM protein PHB1 binds to the OMM protein Nix during mitochondrial dysfunction prior to robust OMM rupture. Additionally, PHB1 is not required for induction of Nix transcription during elevated ROS but is necessary for Nix localization to mitochondria. We nexted determined whether ROS scavengers such as N-acetyl-l-cysteine (NAC) or and mitoTEMPO (mitochondrial-targeted superoxide dismutase mimetic) inhibit PHB1 and Nix interaction. Compared to vehicle-treated control cells, NAC and mitoTEMPO did not alter basal PHB1/Nix interaction by co-immunoprecipitation (Fig. S13). This suggests that dampening of ROS levels does not regulate basal interaction of PHB1 and Nix, at least in this model using ModeK cells.

### PHB1 interaction with Nix is necessary for mitophagy during increased mitochondrial ROS

**Figure 5:**
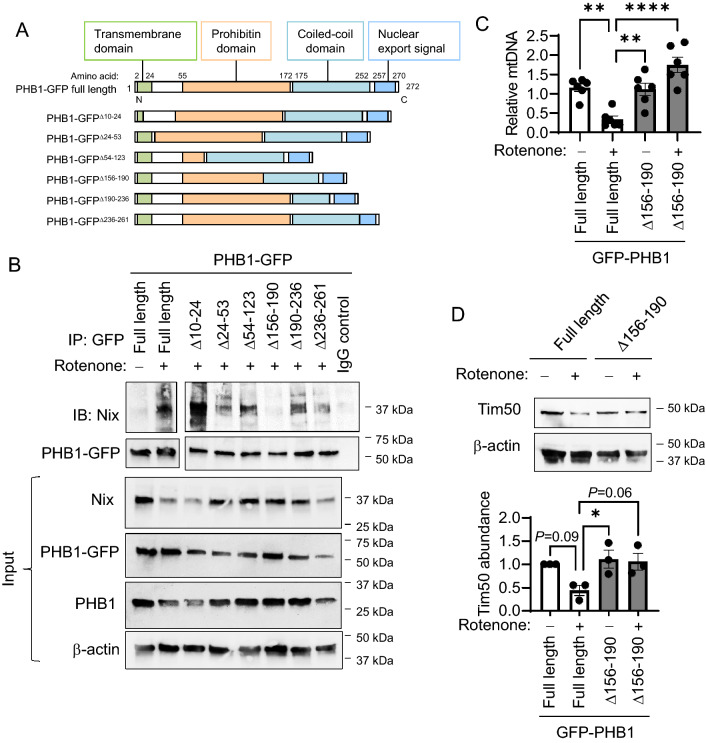
PHB1 interaction with mitophagy receptor Nix is necessary for mitophagy induction by mitochondrial ROS. (**A**) Cartoon depicting PHB1 functional domains and PHB1-GFP deletion constructs. (**B**–**D**) Mode-K cells were transfected with GFP-PHB1 deletion constructs (missing regions denoted as amino acids) and treated with 500 nM rotenone for 2 h. (**B**) Protein was subjected to co-IP of GFP and Nix. Images for IP are from 2 different cropped blots. (**C**) Relative mitochondrial/nuclear DNA level. (**D**) Tim50 protein expression. Results are presented as individual data points ± SEM of 6 (**C**) or 3 (**D**) per treatment group. *P < 0.05, **P < 0.01, ****P < 0.001 by one-way ANOVA and Tukey’s posthoc test. See also Figs. S19–S21 for full-length blots.

To determine binding region of PHB1 crucial for interaction with Nix, Mode-K cells were transiently transfected with full-length PHB1-GFP or PHB1-GFP deletion constructs (Fig. 5A) and treated with rotenone to increase mitochondrial ROS. By co-immunoprecipitation of exogenous PHB1-GFP, PHB1-GFP^Δ156–190^ deletion construct exhibited little-to-no interaction with Nix (Figs. 5B, S19, and S20). Amino acids 156–190 of PHB1 are part of the Prohibitin domain and the coiled-coil domain, both of which are important for protein–protein interactions and project into the mitochondrial inner membranous space^19,21^. Overexpression of PHB1-GFP^Δ156–190^ in Mode-K cells inhibited mitophagy induced by rotenone as demonstrated by sustained mitochondrial DNA:nuclear DNA ratio (Fig. 5C) and sustained Tim50 protein abundance (Figs. 5D, S21). Taken together, these results suggest that PHB1 interaction with Nix is a key inducer of mitophagy during increased mitochondrial ROS.

### PHB1 co-localization with Nix is increased in Crohn’s disease ileal crypts

**Figure 6:**
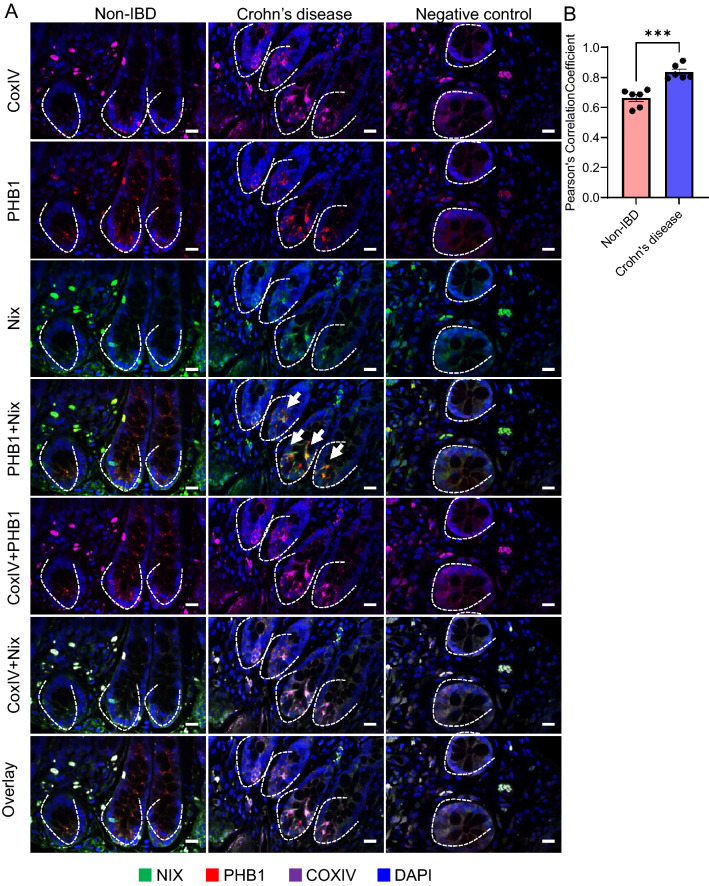
PHB1 interaction with Nix is increased in Crohn’s disease ileal crypts. (**A**) Representative immunofluorescent staining of ileum. Dashed lines outline crypt base. Scale bars: 50 μm. (**B**) Pearson’s correlation coefficient was calculated in 27 ileal crypts/patient across 6 Crohn’s disease patients and 47 ileal crypts/patient across 6 non-IBD control patients. Results are presented as individual data points ± SEM of 6 patients per group. ***P < 0.001 by unpaired, 2-tailed Student’s t test.

PHB1 and NIX co-localization in ileal crypts of Crohn’s disease patients (average 27 ileal crypts/patient across 6 patients) or non-IBD control patients (average 47 ileal crypts/patient across 6 patients) was visualized and quantitated by immunofluorescent staining (Fig. 6A; arrows). PHB1 and NIX co-localization was increased in Crohn’s disease crypts (Fig. 6B), a disease associated with mitochondrial stress^36^.

### Phb1 mediates Nix-induced mitochondrial clearance during reticulocyte maturation

An established role of Nix in mediating mitophagy is in reticulocytes when all mitochondria are eliminated during maturation to erythrocytes^37^. To validate whether Phb1 interaction with Nix is necessary for Nix-induced mitophagy, we crossed *Phb1*^*fl/fl*^ mice with *EpoR*^*Cre*^ mice to generate mice with PHB1 deficiency in cells of the erythroid lineage. 23% of mouse pups were *Phb1*^*fl/*+^, 35% were *Phb1*^*fl/fl*^, 42% were *Phb1*^*fl/*+^:*EpoR*^*Cre*^, and 0% were *Phb1*^*fl/fl*^:*EpoR*^*Cre*^ with equal males and females produced per genotype. Since *Phb1*^*fl/fl*^:*EpoR*^*Cre*^ mice were not viable, all studies utilized *Phb1*^*fl/*+^:*EpoR*^*Cre*^ mice that exhibited 40% loss of Phb1 mRNA and protein expression in reticulocytes (Fig. S22A,B). *Phb1*^*fl/*+^:*EpoR*^*Cre*^ mice exhibited the same body weight gain as control littermates up to 18 weeks of age and had no overt phenotype. To induce erythropoiesis, mice where retroorbitally-bled 2 consecutive days before analysis. *Phb1*^*fl/*+^:*EpoR*^*Cre*^ mice exhibited increased reticulocytes in whole blood (Fig. S22C) and abnormally shaped erythrocytes (Fig. S22D), suggestive of a maturation defect and similar to *Nix*^*−/−*^ mice^37^. To stimulate reticulocyte maturation and concomitant mitochondrial clearance by mitophagy, reticulocytes were purified from whole blood by CD71^+^ MACS magnetic bead/column isolation and cultured for 4 days. Isolated cells were validated as reticulocytes by flow cytometry (Fig. S23A). *Phb1*^*fl/*+^:*EpoR*^*Cre*^ reticulocytes exhibited defective mitochondrial clearance as visualized by retained mitoTracker Red staining after 4 days of culture (Fig. S23B). Collectively, these results suggest that Phb1 deficiency in reticulocytes inhibits mitochondrial clearance by mitophagy, phenocopying *Nix*^*−/−*^ reticulocytes^37^.

## Discussion

Our results demonstrate that much like PHB2, PHB1 acts as a sensor for stress localized to the IMM and matrix to induce mitophagy. Unlike PHB2, PHB1 acts independently of Parkin as a mitophagy inducer via Nix in response to increased ROS not requiring OMM rupture. We speculate that PHB1 and PHB2 serve complimentary functions in the induction of mitophagy with PHB1 acting independently of Parkin during mitochondrial stress (increased ROS) and PHB2 acting in a Parkin-dependent manner as reinforcement upon OMM rupture. This dual mechanism of mitochondrial incorporation into the autophagy pathway may enhance the efficiency of removal of damaged mitochondria.

Membrane rupture was shown to be a necessary event for sequestration of organelles such as lysosomes and endosomes into the autophagy degradation pathway^38,39^. In contrast, our results indicate that mitochondria can be removed by mitophagy prior to OMM rupture via Nix. Nix localizes to mitochondria and endoplasmic reticulum and is a key mediator of mitophagy in cardiomyocytes and in anucleated cells such as reticulocytes and platelets^40–43^. Here, we demonstrate that reticulocytes deficient in Phb1 expression phenocopy *Nix*^*−/−*^ reticulocytes with defective mitochondrial clearance, suggesting Phb1 is a crucial regulator of Nix-induced mitophagy. Expression of Nix is controlled by the transcription factor hypoxia-inducible factor 1α (Hif1α), suggesting hypoxia is a central activator of Nix-induced mitophagy^44–46^. Low levels of elevated mitochondrial ROS participate in hypoxia response by regulating the stability of Hif1α, whereas high levels of mitochondrial ROS induce OMM rupture, facilitating apoptosis via release of mitochondrial proteins into the cytoplasm such as Cytochrome C^18^. In this way, PHB1 may dampen mitochondrial-mediated apoptosis via Nix-induction of mitophagy prior to severe mitochondrial damage. In the intestinal epithelium, Nix is increased in inflammatory bowel disease patients and in murine colitis in a manner dependent on mitochondrial ROS^45^. *Nix*^*−/−*^ mice are more susceptible to chemically- or adoptive T cell transfer-induced colitis^45^, supporting an important role of Nix-induced mitophagy in the protection against intestinal inflammation. Our results in *Phb1*^*iΔIEC*^ mice suggest that *Phb1* deletion with compensatory deficiency in PHB2 inhibit mitophagy involved in the development of Crohn’s disease-like ileitis^29^. It is well-established that goblet and Paneth cells are susceptible to autophagy inhibition^7,8^; our results demonstrate a specific role of mitophagy in these cells and in maintaining intestinal homeostasis. PHB1 interaction with Nix may be important in cells highly susceptible to mitochondrial dysfunction, such as Paneth cells^29^, as a first line of defense to remove stressed mitochondria. In our previous publication, we demonstrated that the mitochondrial-targeting antioxidant mitoTEMPO could ameliorate ileitis in *Phb1*^*iΔIEC*^ mice, suggesting that mitochondrial-derived ROS contributed to the pathology of this model^29^. Given the results presented here, we speculate that accumulation of damaged mitochondria due to inefficient mitophagy in *Phb1*^*iΔIEC*^ mice also contribute to the pathology of this model. Future studies will test whether induction of mitophagy, such as via Urolithin A treatment^47^, can dampen ileitis severity in *Phb1*^*iΔIEC*^ mice.

The exact mechanism(s) of how PHB1 interaction with Nix facilitates Nix mitochondrial localization or Nix binding with LC3II is not known. PHB1 exhibits chaperone activities, stabilizing mitochondrial proteins for optimal function^23,24^. PHB1 may exert similar stabilizing function for Nix in the mitochondria. Although only 11 amino acids of the Nix C-terminus localizes to the intermembrane mitochondrial space, this C-terminus has been shown to be an important regulator of mitophagy^48^. Key C-terminal amino acid residues of Nix that extend into the mitochondrial intermembranous space are responsible for proper Nix-dependent mitophagy^48^. A large C-terminal proportion of PHB1 (amino acids 28–272) reaches into the intermembrane mitochondrial space facilitating protein–protein interactions via the Prohibitin domain and coiled-coil domains^19–21^. Our studies suggest portions of the PHB1 C-terminus are necessary for interaction, either directly or indirectly, with Nix. Interaction of Prohibitins with the C-terminus of OMM-localized proteins, such as Bif-1, Miro1, and Miro2, has been previously reported^22,49^. During mitochondrial swelling and unfolding of the IMM, the IMM can become juxtaposed to the OMM which may further facilitate interaction between proteins residing in these membranes^50^. It has also been demonstrated that post-translational modifications of Nix regulate its pro-mitophagy activity, with increased affinity for LC3II by C-terminal phosphorylation of Nix within the LIR domain^51^, dimerization of Nix, and subsequent mitophagy initiation^48^. Future studies will determine whether interaction of Nix with PHB1 are necessary for its phosphorylation and subsequent LC3II binding and/or dimerization, providing an additional layer of mitophagy regulation.

PHBs serve multiple, diverse cellular functions including regulation of cell-cycle progression, senescence, gene transcription, apoptosis, and mitochondrial fitness^26^. Global genetic deletion of *Phb1* or *Phb2* results in embryonic lethality in mice, suggesting an essential role in early development^30,52,53^. Although PHB1 and PHB2 function primarily in the mitochondria, independent functions of PHB1 thus far distinct from PHB2 include inhibition of Wnt signaling, interaction and regulation of SH2 proteins (Shp1, Lyn, Syk), and interaction with Ras, C-RAF, and Akt in plasma membrane lipid rafts^33^. PHB2 has been shown to regulate ERα signaling and interact with LC3II^33^. Decreased PHB1 and PHB2 expression is associated with aging and age-associated diseases with known mitophagy impairment including Parkinson’s disease and Alzheimer’s disease^54,55^. *Phb2* deletion specifically in forebrain neurons results in neurodegenerative disease and premature death in mice^56^. The opposite is noted in many types of cancer with increased PHB1 and PHB2 expression^57^ and mitophagy serving context-dependent tumor suppressor and tumor promoter functions^58^. We previously showed that PHB1 knockdown in the colorectal cancer cell lines Caco2 and HCT-116 induced mitochondrial stress and mitophagy^59^ in contrast to our current findings in non-transformed IECs. We speculate that this is due to mitochondrial and metabolic adaptations during transformation with additional mechanisms of mitophagy not dependent on PHB1. In terms of inflammation, PHB1 has been shown to be protective across numerous tissue types including the intestine, liver, heart, pancreas, lung, and brain^60–62^ whereas this is less studied for PHB2. Given the established role of mitophagy in aging, neurodegeneration, cancer, and inflammation, the functions of PHB1 and PHB2 in mitophagy likely play a mechanistic role in these diseases. Using the gastrointestinal epithelium as a model system highly susceptible to autophagy inhibition, we reveal a role of PHB1 in direct binding of Parkin-independent mitophagy induction and intestinal homeostasis. PHB1 has pleiotropic roles in mitochondrial quality control, simultaneously regulating mitochondrial function and stimulating mitophagy induced by increased mitochondrial ROS.

## Methods

### Mouse models

*Phb1*^*fl/fl*^ mice (previously described^63^) were crossed with *Villin-Cre-ER*^*T2*^ mice, both on the C57Bl/6 genetic background. 8-week old male and female *Phb1*^*fl/fl*^ and *Phb1*^*fl/fl*^*:Villin*-CreERT2 (referred to as *Phb1*^*iΔIEC*^ mice^29^) were intraperitoneally injected with 100 μl of 10 mg/ml tamoxifen (Sigma Aldrich) as previously described^64^ for 4 consecutive days to induce deletion of *Phb1* from IECs. Mice were sacrificed 1 week after the first tamoxifen injection. *Parkin*^*−/−*^ mice were obtained from The Jackson Laboratory (#006582). *EpoR*^*Cre*^ mice were obtained from The Jackson Laboratory (#035702) and were crossed to *Phb1*^*fl/fl*^ mice. To induce erythropoiesis, 350 μl of blood was removed by orbital bleed over 2 consecutive days from EpoR^Cre^:*Phb1*^*fl/*+^ and *Phb1*^*fl/*+^ mice. Experiments were performed with age- and gender-matched littermate mice. All mice were grouped-housed in standard cages under SPF conditions and were allowed standard chow and tap water ad libitum. All experiments were approved by the University of Colorado Anschutz Medical Campus Institutional Animal Care and Use Committee and our reporting follows the recommendations in the ARRIVE guidelines. All methods were carried out in accordance with relevant guidelines and regulations.

### Human patient mucosal biopsy immunofluorescent staining

The study design and conduct were authorized by the Baylor University Medical Center, Dallas, Texas IRB committees (IRB 016-083) and complied with all relevant regulations and guidelines regarding the use of human study participants. The study was conducted in accordance with the criteria set by the Declaration of Helsinki. Informed consent was obtained from all subjects. Crohn’s disease patients and non-IBD controls were recruited from Baylor University Medical Center during endoscopy procedure to obtain mucosal biopsies from the terminal ileum. Non-IBD controls underwent an elective endoscopy for clinical purposes, did not have a history of IBD, and lacked endoscopic and histological inflammation. Biopsies were immediately formalin-fixed and later embedded in paraffin. Immunofluorescent staining was performed as described below. Sections were visualized using Zeiss Axioskop Plus microscope. Co-localization of PHB1 and NIX was analyzed by Pearson’s correlation coefficient using Fiji (ImageJ). Briefly, immunofluorescent images were converted to 8- or 16-bit, region of interest selected in one of the channels, and chose “Coloc2” plugin from “Colocalization” under “Analyze” to find the value for the Pearson’s correlation coefficient.

### Cell lines and transfection

Mode-K cells were acquired from Inserm, France. Hela cells were purchased from the American Type Culture Collection (ATCC). Cells were cultured in 1× Dulbecco’s modified Eagle’s medium (DMEM) supplemented with 10% fetal bovine serum and 40 mg/L penicillin and 90 mg/L streptomycin. Cells were maintained in an incubator with 5% CO_2_ at 37 °C. All experiments were performed on Mode-K cells between passages 21 and 30. All cell lines were verified mycoplasma free using Genlantis MycoScope PCR Detection kit (Fisher Scientific) 1 week prior to experiment start and every 3 months thereafter. Cells were treated with 500 nM rotenone (Sigma-Aldrich), 2.5 μM oligomycin (Sigma-Aldrich), 100 nM antimycin A (Sigma-Aldrich), 10 mM N-acetyl-l-cysteine (Sigma Aldrich), or 25 nM mitoTempo (Sigma-Aldrich).

To knockdown PHB1 expression, cells were transiently transfected using Nucleofector T kit (Lonza) with Stealth RNAi™ against *Phb1* (5′-CAGAAUGUCAACAUCACACUGCGCA -3′, Invitrogen, referred to as siPhb1a herein), a second pooled siPhb1 (FlexiTube GeneSolution GS5245, Qiagen, referred to as siPhb1b herein), or Stealth RNAi™ siRNA Negative Control Med GC (Invitrogen) at 20 µm concentration for 48 h. Mouse PHB2 overexpression plasmid (MR218376, Origene), pSELECT-GFP-LC3 expression plasmid (#psetz-gfplc3, Invivogen), mito-RFP-EGFP expression plasmid^31^, or pGL3-Nix^65^ (a generous gift from Dr. Gerald Dorn, Washington University, St. Louis) were transfected into Mode-K cells using Nucleofector T kit. To quantitate mito-RFP-EGFP expression, images were captured from EVOS fluorescent microscope. Quantitation of images was performed with ImageJ software for yellow and red pixel intensity using the average of 50 cells per well.

### Antibodies

The following antibodies were used for western immunoblotting: PHB1 (70R-5543, Fitzgerald), PHB2 (14085, Cell Signaling), LC3 (L7543, Sigma), p62 (5114, Cell Signaling), Tim50 (sc-515268, Santa Cruz), CoxIV (4850, Cell Signaling), Nix (12396, Cell Signaling), FundC1 (ABC506, Millipore), Bnip3 (3769, Cell Signaling), Optineurin (100000, Cayman Chemical), NDP52 (H000 10241-B01P, Abnova), GST (sc-138, Santa Cruz), Parkin (MAB5512, Millipore), GFP (2956, Cell Signaling), Cytochrome C (4280, Cell Signaling), β-actin (A1978, AC-15, Sigma-Aldrich), β-tubulin (T4026, Sigma Aldrich). Antibodies were validated by western blot using the respective recombinant protein as positive control. The following antibodies were used for immunostaining: LC3 (L7543, Sigma), Lysozyme (sc27956, Santa Cruz), Muc2 (ab134119, EPR6145, Abcam), CoxIV (4850, Cell Signaling), PHB1 (70R-5543, Fitzgerald), and Nix (12396, Cell Signaling). Isotype controls were included to validate immunostaining.

### Isolation of intestinal epithelial cells (IECs)

IEC isolation was performed as previously described^66^.

### Transmission electron microscopy

Distal ileum or Mode-K cells were fixed in 2% gluteraldehyde in 1× PBS, dehydrated and embedded in epoxy resin for electron microscopy. Ultrathin 70 nm sections were examined on a transmission electron microscope (Hitachi BioMedical TEM).

### Immunofluorescent staining

7 μM paraffin-embedded sections of mouse or human ileum were dehydrated in xylene and ethyl alcohol gradient, incubated in 10 mM sodium citrate for antigen epitope retrieval, blocked in 5% normal donkey serum, and exposed to the following primary antibodies at 4 °C overnight: PHB1 (anti-mouse; 1:500; MS-261-P1, Neomarkers), NIX (anti-rabbit; 1:1000; 12396, Cell Signaling), and CoxIV (anti-goat; 1ug/ul; LSB3256, LSBio). After washing in 1 × PBS, sections were incubated with secondary antibodies (Cy5-conjugated AffiniPure donkey anti-mouse IgG (715-175-150, Jackson ImmunoResearch), FITC-conjugated AffiniPure F(ab’)_2_ Fragment donkey anti-rabbit IgG (711-096-152, Jackson ImmunoResearch), and Cy3-conjugated AffiniPure donkey anti-goat IgG (705-165-003), Jackson ImmunoResarch). Sections were then stained with 4′,6-Diamidino-2-phenylindole dihydrochloride (DAPI, D9542, Sigma) at 1:1000 in 1× PBS for 1 min. Sections were visualized using Zeiss Marianas 3i confocal microscope.

### Immunohistochemical staining

The following antibodies were used for immunostaining: PHB1 (70R-5543, polyclonal, Fitzgerald), 7 μM paraffin-embedded sections of ileum were dehydrated in xylene and ethyl alcohol gradient, incubated in 0.3% H_2_O_2_ for 30 min, washed, incubated in 10 mM sodium citrate for antigen epitope retrieval, blocked in 5% normal goat serum, and exposed to PHB1 antibody (70R-5543, polyclonal, Fitzgerald) at 1:500 dilution at 4 °C overnight. Peroxidase-labeled anti-rabbit immunoglobulin G secondary antibody and ABC reagent were added using the peroxidase-conjugated avidin ABC kit (Vector Laboratories, Burlingame, CA). Sections were counterstained with hematoxylin to visualize histology.

### Detection of mitochondrial DNA versus nuclear DNA

Total DNA (genomic and mitochondrial) was isolated using DNeasy Blood and Tissue Kit (Qiagen). The amount of mitochondrial DNA was measured by quantitative real-time PCR (qRT-PCR) using primers for the mitochondrial encoded gene *mt-Co1*: sense: 5′-CTGAGCGGGAATAGTGGGTA-3′; antisense: 5′-TGGGGCTCCGATTATTAGTG-3′^67^. This was compared to the amount of nuclear DNA using *18S* primers: Sense: 5′-CCCCTCGATGCTCTTAGCTGAGTGT-3′; antisense: 5′-CGCCGGTCCAAGAATTTCACCTCT-3′.

### Western blot analysis

Total protein was isolated from whole ileum or isolated IECs and separated by SDS-PAGE and analyzed by western blotting as described previously^68^. Mitochondrial and cytosolic extracts were isolated using mitochondrial isolation buffer (70 mM sucrose, 220 nM D-mannitol, 2 mM Hepes), followed by centrifugation at 900×*g* for 10 min. The supernatant was centrifuged at 10,000×*g* for 10 min to pellet mitochondria.

Mitophagic flux was determined during Bafilomycin A1 treatment as described by Viva Detect Autophlux kit (VB3000, Viva Bioscience). Briefly, Tim50 and CoxIV were measured by western blotting, and densitometric units of bands were obtained using Photoshop and normalized to β-actin band intensity. Control (Naïve and siNC) mitophagic flux (Control MF) was calculated using normalized densitometric units: Control MF = (Control + BafA) – (Control – BafA). siPHB1 mitophagic flux (siPHB1 MF) = (siPHB1 + BafA) – (siPHB1 – BafA). The change in mitophagic flux (ΔMF) between control and PHB1 knockdown was calculated as: ΔMF = siPHB1 MF – Control MT.

For co-immunoprecipitation experiments, 1 mg pre-cleared total protein was incubated with 1 μg antibody/mg protein (GFP (2956, Cell Signaling), PHB1 (70R-5543, Fitzgerald), Nix (12396, Cell Signaling), IgG negative control (3900, Cell Signaling)) at 4 °C for 16 h. Protein/antibody mixtures were then incubated with Protein G Plus-Agarose beads (sc-2002, Santa Cruz) at 4 °C for 2 h. After precipitation of beads and bead-bound protein complexes by centrifugation, bound proteins were eluted by denaturating buffer and visualized by SDS-PAGE and western blotting.

### Digital image acquisition

Chemiluminescence pictures of western blots were taken using BioRad VersaDoc gel documentation system and quantified by QuantityOne software (Version 4).

### GST pulldown assay

Full-length coding region of PHB1 was cloned into the pGEX-6p-1 vector (Addgene) and transformed into BL21(DE3) cells that were then treated with IPTG to induce expression of fusion proteins. pGEX-6p-1 empty vector was used as a negative control. GST fusion proteins (bait protein) were isolated via lysis of bacterial pellets and immobilized onto glutathione agarose beads. 500 μg protein from primary mouse IECs or Mode-K cells (prey protein) was incubated with the GST-protein loaded agarose beads at 4 °C for 2 h. Beads were then pelleted by centrifugation, washed 3 times with GST pull down wash buffer (2 mM HEPES-HCl, 150 mM NaCl, 1 mM EDTA, 0.5% Tween), boiled in Laemmli buffer/β-mercaptoethanol, and visualized by SDS-PAGE.

### PHB1-GFP deletion constructs

The generation of pEGFPN1-PHB1 was previously described^59^. Deletion constructs were generated by 2 separate PCR reactions using pEGFPN1-PHB1 full length as template, high fidelity Taq (Invitrogen), and primers shown in Table 1. One primer in each pair for the first PCRs contain a ~ 20 bp complementary region that allows the fusion of the two domain fragments in the second PCR reaction. The product from the second PCR reaction was ligated into pEGFPN1 vector (Clontech) using the Quick Ligation Kit (New England Biolabs) via *Hind*III and *Bam*HI sites. All deletion constructs were verified by Sanger sequencing.

**Table 1:** Primers used for PCR generation of PHB1-GFPN1 deletion constructs.

Deletion construct	Primers, first PCR amplification		Primers, second PCR amplification
	Pair	(Complementary sequence underlined)	
PHB1-GFP^Δ10–24^	1	Sense: Primer 1 Antisense: 5′-CACATTATATAAGGCAGAGCCAATGGACTCAAA-3′	Sense: Primer 1 Antisense: Primer 2
	2	Sense: 5′-TCTGCCTTATATAATGTGGATGCTGGGCACAGAGCT-3′ Antisense: Primer 2	
PHB1-GFP^Δ24–53^	1	Sense: Primer 1 Antisense: 5′-CGGGATGAGAAAATGAGTGTTCACCACGCCTCCTGC-3′	Sense: Primer 1 Antisense: Primer 2
	2	Sense: 5′-ACTCATTTTCTCATCCCGTGGGTACAGAAACCAAT -3′ Antisense: Primer 2	
PHB1-GFP^Δ54–123^	1	Sense: Primer 1 Antisense: 5′-CCACGGGATGAGAAAATGAGTCCCTTCCCCTACCAC-3′	Sense: Primer 1 Antisense: Primer 2
	2	Sense: 5′-CATTTTCTCATCCCGTGGCGCATCTTCACCAGCATCGGA-3′ Antisense: Primer 2	
PHB1-GFP^Δ156–190^	1	Sense: Primer 1 Antisense: 5′-GGCCCTCTCTGCTTCCTGCTCTGTAAGGTCGTCGCT-3′	Sense: Primer 1 Antisense: Primer 2
	2	Sense: 5′-CAGGAAGCAGAGAGGGCCAGATTTGTGGTGGAAAAG-3′ Antisense: Primer 2	
PHB1-GFP^Δ190–236^	1	Sense: Primer 1 Antisense: 5′-TTCCAGCTTGCGCAGCTCCTGAGCCACCTGTTTGGC-3′	Sense: Primer 1 Antisense: Primer 2
	2	Sense: 5′-GAGCTGCGCAAGCTGGAAGCTGCAGAGGACATCGCG-3′ Antisense: Primer 2	
PHB1-GFP^Δ236–261^	1	Sense: Primer 1 Antisense: 5′-GATCAGGCCATCCCCTGCAGTGGCCAGTGAGTTGGC-3′	Sense: Primer 1 Antisense: Primer 2
	2	Sense: 5′-GCAGGGGATGGCCTGATCGCGGGGCAGTCCGTGCTC-3′ Antisense: Primer 2	

	Primer 1 (sense)	Primer 2 (antisense)	
PHB1 primers	5′-CGCGCCAAGCTTATGGCTGCCAAAGTG-3′ *Hind*III site underlined	5′-AATTGGATCCCCTCCCTGGGGCAGCTGGA-3′ *Bam*HI site underlined	

### Luciferase reporter assay

Mode-K cells were co-transfected with pGL3 or pGL3-Nix, siNC or siPHB1, and pRL-CMV (Renilla luciferase as internal control; E2231, Promega). 72 h post-transfection, cells treated with 500 nM rotenone for 2 h. Luciferase activity was measured using the Dual-Luciferase Reporter Assay system (E1910, Promega) and a BioTek luminometer. Relative luciferase was calculated by normalizing firefly luciferase activity to renilla luciferase activity.

### mitoSOX measurements

Mode-K cells were incubated with Hank’s balanced salt solution (HBSS) with 5 μM MitoSOX Red Mitochondrial Superoxide Indicator dye (Invitrogen) for 10 min at 37 °C. Cells were washed twice with warm HBSS and fluorescent intensity was measured at 510 nm excitation/580 nm emission.

### Complete blood count (CB) analysis

Whole blood was collected into K_2_-EDTA tubes and analyzed for CBC with 3 part differential and reticulocyte count by the Comparative Pathology Shared Resources Core, University of Colorado Anschutz Medical Campus.

### Wright Giemsa staining

Whole blood was streaked thin across a glass slide and allowed to air-dry. The slide was then stained with Wright-Giemsa (9990710, Fisher Scientific) for 2 min and rinsed in Wright-Giemsa Buffer solution for 2 min.

### Reticulocyte isolation

Peripheral blood mononuclear cells (PBMCs) were isolated by Ficoll-Paque Plus (95021-205, VWR) gradient. PBMCs were resuspended in Miltenyi MACS buffer (1:20 dilution of MACS BSA stock solution (130-091-376, Miltenyi) and AutoMACS Rinsing solution (130-091-222, Miltenyi)) and passed through Miltenyi pre-separation filter (130-041-407) to obtain single cell suspension. 2 million cells were labeled with CD71 biotin antibody (130-109-572, Miltenyi) for 10 min at 4 °C, washed in MACS buffer, and exposed to anti-biotin microbeads (130-090-485, Miltenyi) for 15 min at 4 °C. The microbead solution was then passed through MS columns (130-042-201, Miltenyi)/MiniMACS separator (130-042-102, Miltenyi) as described by the manufacturer to isolate reticulocytes via CD71^+^ expression.

### Reticulocyte culturing

CD71^+^ MACS isolated reticulocytes were cultured in Iscoves modified Dulbecco medium (IMDM, 12440053, Gibco) with 30% heat-inactivated fetal bovine serum, 1% bovine albumin, 100 units/ml penicillin, 100 ug/ml streptomycin (Sigma), and 0.1 mM α-thioglycerol (M6145, Sigma). Fresh media was added every 2 days.

### Quantitative real-time PCR

Total RNA was isolated from reticulocytes using Trizol (ThermoFisher). Quantitative real-time PCR was performed as described previously^68^. For graphical representation of quantitative PCR data, the ∆∆C_T_ was calculated as follows: ∆∆C_T_ = (Ct, _target_ − Ct, _β-actin_)_*Phb1fl/*+:*EpoRCre*_ − (Ct, _target_ − Ct, _β-actin_) _*Phb1fl/*+_, with the final graphical data derived from 2^−∆∆CT^. Primers sequences: *Phb1* sense: 5′-TAAGACTGGGTCCTGCCATT-3′; *Phb1* antisense 5′-GTGCTTGCATCAGAGTCAGG-3′.*β-actin* sense: 5′-TATGCCAACACAGTGCTGTCTGG-3′; *β-actin* antisense 5′-TACTCCTGCTTGCTGATCCACAT-3′.

### MitoTracker staining

Cultured reticulocytes were exposed to 100 nM MitoTracker RedCMXRos (9082, Cell Signaling) in culture media for 20 min. Unfixed cells were visualized using Zeiss Axioskop Plus microscope.

### Flow cytometry

Whole blood was removed by retroorbital bleed and stained for flow cytometry (BD FACSCanto II) using the following antibodies or fluorescent dyes: Biotin anti-mouse CD71 REAfinity (130-109-572, Miltenyi), anti-biotin-PE (130-113-291, Miltenyi), 0.0025 µg/µL PE-Cyanine7 Anti-Mouse TER-119/Erythroid Cells (116222, BioLegend), 0.0025 µg/µL APC-Cyanine7 Anti-Mouse CD45 (103116, BioLegend), 100 nM Thiazol orange (390062, Sigma Aldrich), 500 nM MitoTracker Deep Red FM (8778, Cell Signaling), and 2.5 µg/mL 7-AAD live-dead viability staining (420404, BioLegend).

### Statistical analysis

Distinct sets of *Phb1*^*iΔIEC*^ and *Phb1*^*fl/fl*^ mice were used for histological analysis (immunofluorescent staining, TEM; n = 8 per group) or biochemical assays (western blot, mtDNA; n = 8 per group). For starvation experiments, measurements were taken from a distinct set of 7 mice per treatment group. For cell culture experiments, measurements were taken from distinct samples. Covariates were not tested. Sample size was calculated as follows: expecting a 97% rate of *Phb1* knockout, and setting a twofold increase in Tim50 protein expression as a biologically relevant response to basal *Phb1* deletion, with an alpha and beta level set at 0.05 and 0.2, respectively, a sample size of n = 8 mice of each genotype was required (Stata version 9, StataCorp). For western analysis on mouse samples, all samples were included unless protein sample was exhausted on previous blots. For other measurements, no data was excluded. Animals were allocated to experimental groups based on genotype and to assign even numbers of males and females, no further randomization was used. Investigators were blinded to group allocation during data analysis. All findings for basal alterations in *Phb1* deficient mice were replicated in non-starved mice. All cell experiments were performed in at least 2 separate experiments. All data were collected using Microsoft Excel 2018. Values are expressed as mean ± SEM or as individual data points ± SEM. Statistical analysis was performed using unpaired 2-tailed Student’s *t* test for single comparisons, one-way ANOVA with Tukey’s post-hoc test for multiple comparisons, or two-way ANOVA with Tukey’s post-hoc to test for the interaction of genotype and treatment (starvation or rotenone) (PRISM 8.2, GraphPad Software). *P* < 0.05 was considered significant.

## Supplementary Information

Supplementary Figures.

## Data Availability

The datasets used and/or analysed during the current study available from the corresponding author on reasonable request.

## Abbreviations

ETC: Electron transport chain
IEC: Intestinal epithelial cell
IBD: Inflammatory bowel diseases
IMM: Inner mitochondrial membrane
OMM: Outer mitochondrial membrane
LIR: LC3-interacting region
PHB: Prohibitin
ROS: Reactive oxygen species
