# Supplementary material for: Inner mitochondrial membrane protein Prohibitin 1 mediates Nix-induced, Parkin-independent mitophagy
Source: Sci Rep. 2023 Jan 2;13:18. doi: 10.1038/s41598-022-26775-x (PMC9807637; doi:10.1038/s41598-022-26775-x)
Supplement: Supplementary file 1 — Supplementary Figures. [file 41598_2022_26775_MOESM1_ESM.pdf]

Supplemental Figure S1

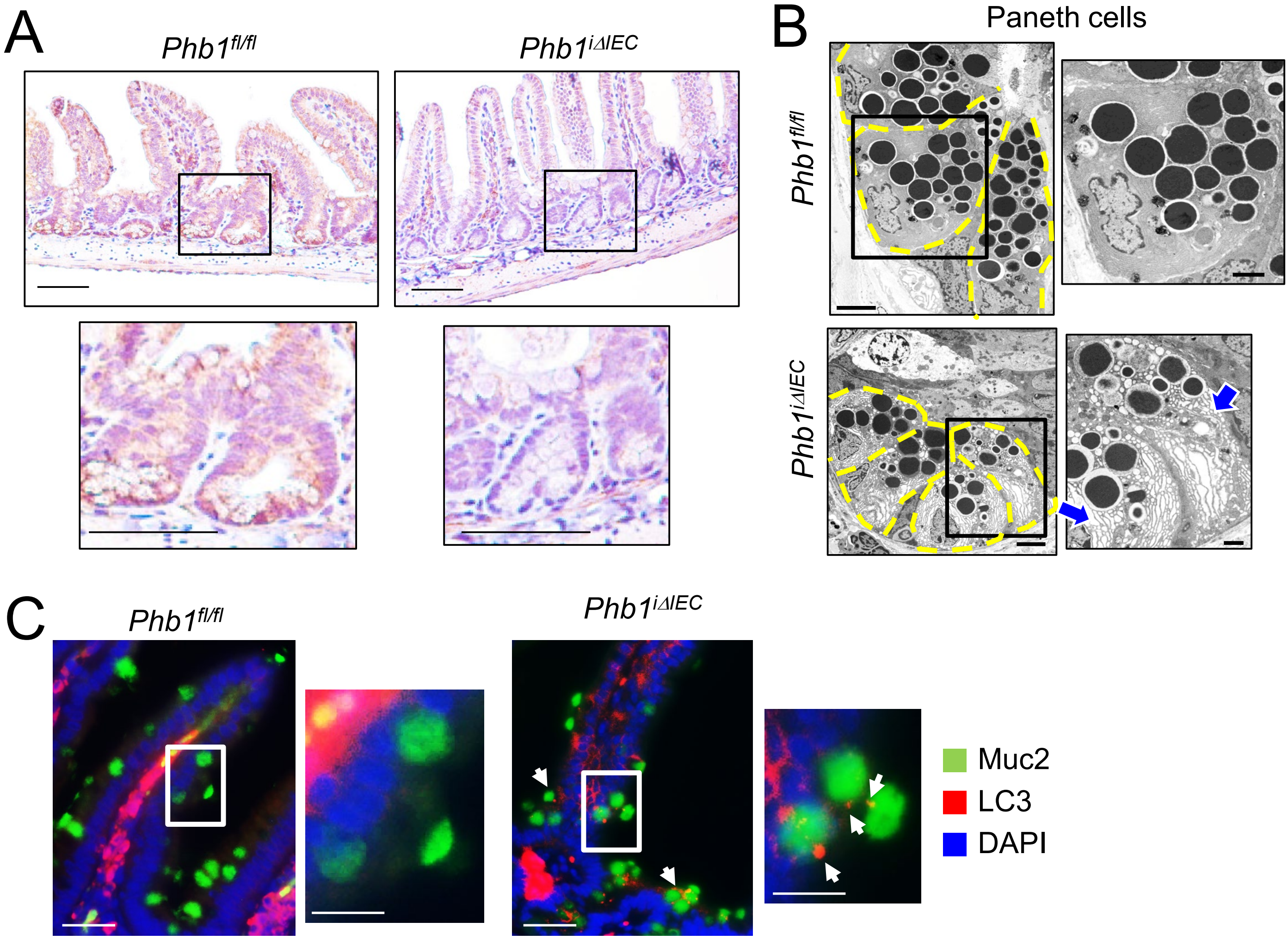

Figure S1. *Phb1$^{i\Delta IEC}$* mice exhibit alterations in intestinal Paneth and goblet cells. (A) PHB1 immunohistochemistry staining of mouse ileum. Scale bars: 250 µm, boxed pullout: 75 µm. (B) TEM of Paneth cells (yellow outline) in ileum crypt base. *Phb1$^{i\Delta IEC}$* Paneth cells have decreased electron-dense secretory granules and vesiculate ER (arrows). Scale bars: 4 µm, boxed pullout: 2 µm. (C) LC3 puncta in goblet cells marked by Muc2^{+} staining (arrows). Scale bars: 50 µm, boxed pullout: 20 µm.

Supplemental Figure S2

Uncropped immunoblots in Fig. 1D

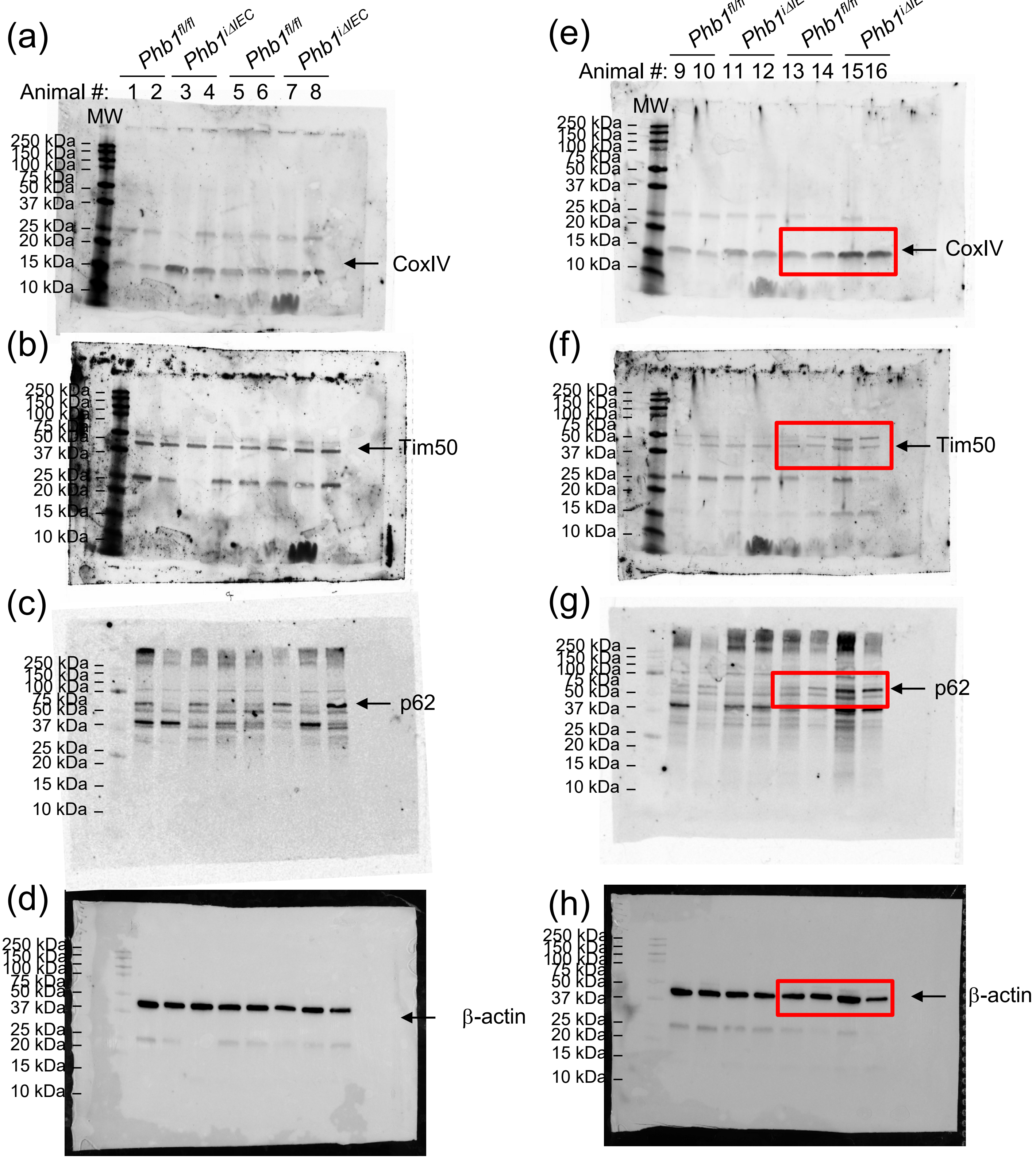

Figure S2. Original uncropped immunoblots. Immunoblots for (a, b) PHB2 and (c, d) β-actin in isolated intestinal epithelial cells from *Phb1*$^{fl/fl}$ or *Phb1*$^{i\Delta IEC}$ mice. Antibodies in (a, b, d, e, f, h) were probed using mouse fluorescent secondary in Dylight 650 channel and (b, f) used rabbit fluorescent secondary in Dylight rabbit channel. (a-d) and (e-h) were probed on the same blot. Each lane represents IECs isolated from an individual mouse. MW, molecular weight. Red boxes indicate regions that were cropped and presented in figures within the manuscript.

Supplemental Figure S3

Uncropped immunoblots in Fig. 2A top panel

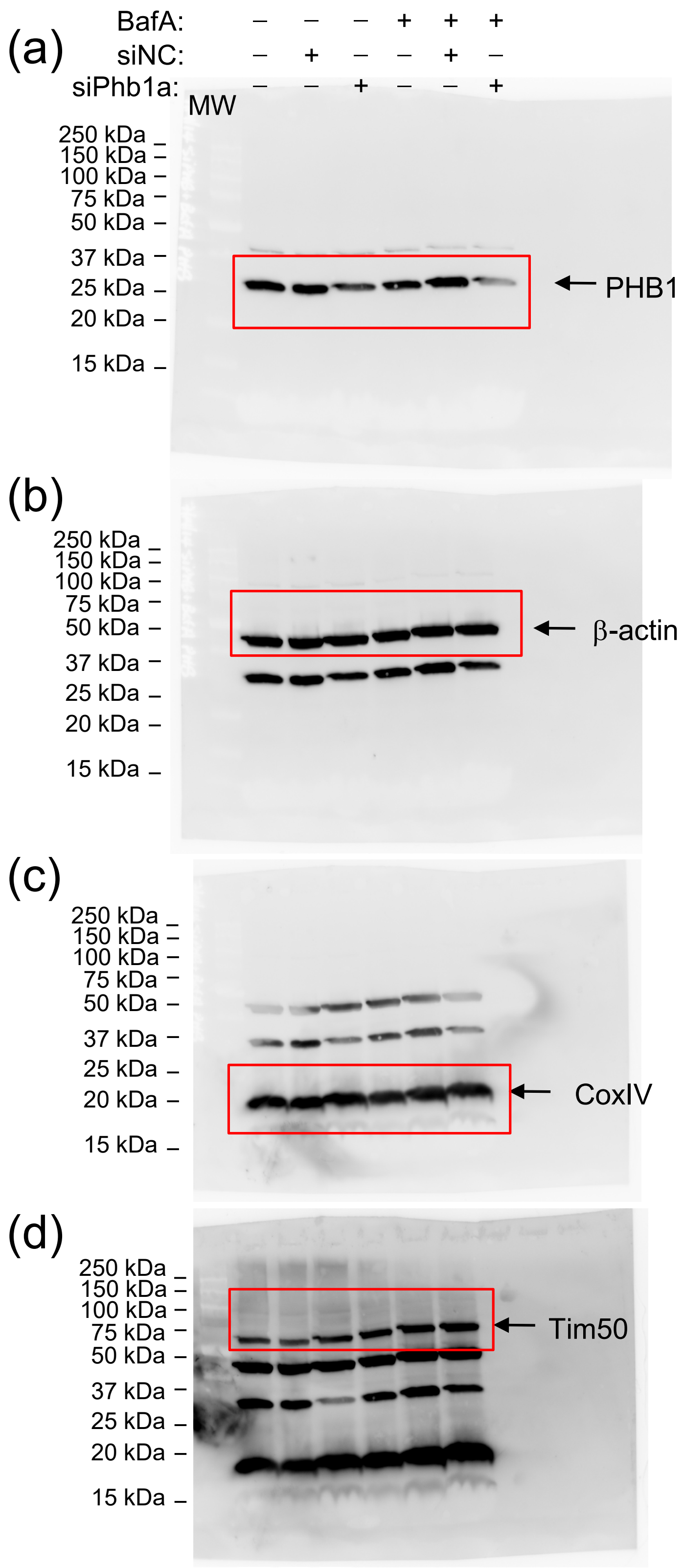

Figure S3. Original uncropped immunoblots. Imunoblots for (a) PHB1, (b) β-actin, (c) CoxIV, (d) Tim50 in siPhb1 knockdown or siNC (control) Mode-K cells treated with BafA or veh. All antibodies were probed on the same blot in the following order: PHB1, β-actin, CoxIV, Tim50. MW, molecular weight. Red boxes indicate regions that were cropped and presented in figures within the manuscript.

Supplemental Figure S4

Uncropped immunoblots in Fig. 2A bottom panel

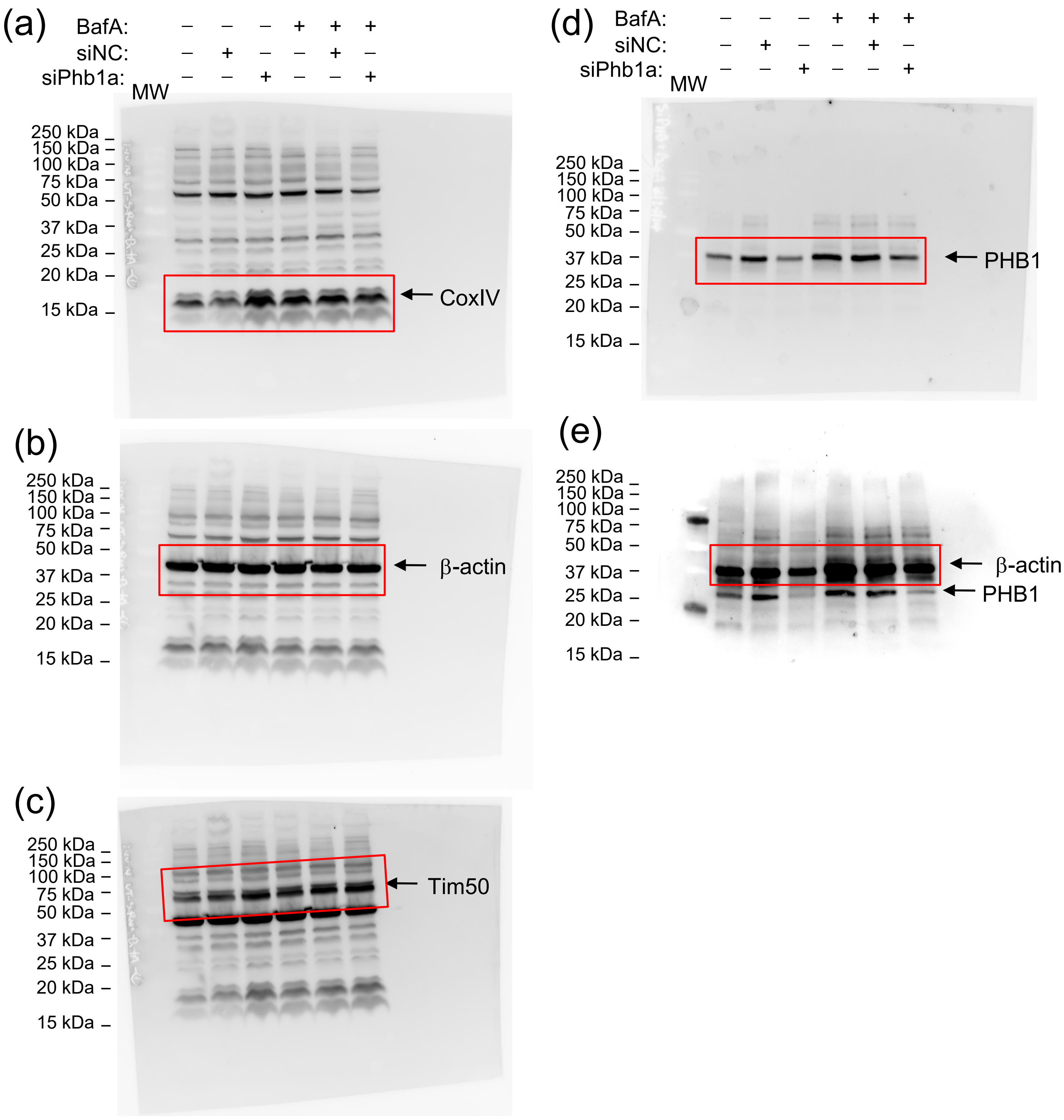

Figure S4. Original uncropped immunoblots. Immunoblots for (a) CoxIV, (b) β-actin, (c) Tim50, (d) Phb1, and (e) β-actin in siPhb1 knockdown or siNC (control) Mode-K cells treated with BafA or veh. Antibodies in (a), (b), and (c) were probed on the same blot. PHB1 (d) was probed on a separate blot and normalized to β-actin run on the same blot in (e) to prevent band overlap. MW, molecular weight. Red boxes indicate regions that were cropped and presented in figures within the manuscript.

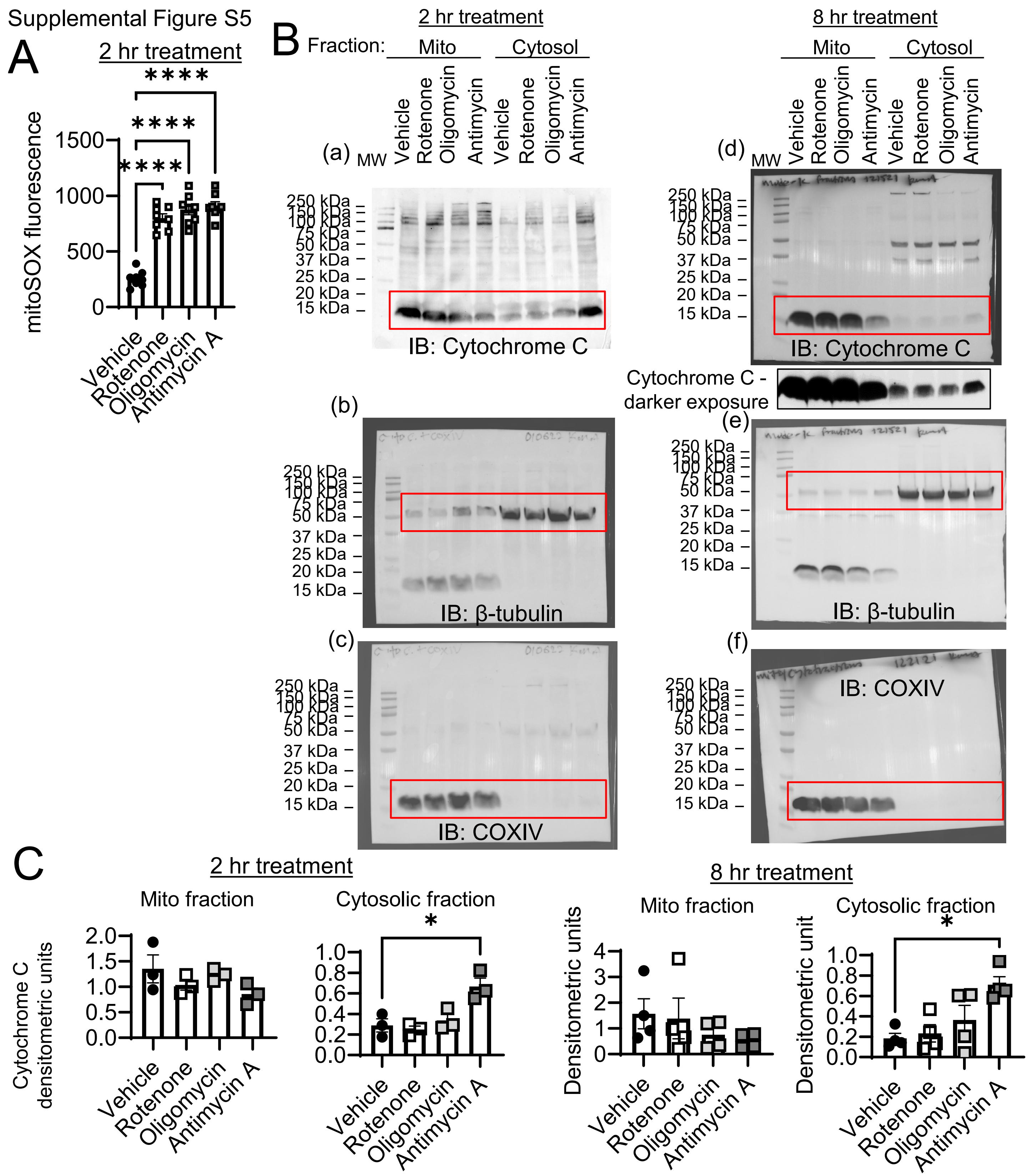

Figure S5. Oligomycin and antimycin A induce robust OMM rupture. Mode-K cells were treated with 500 nM rotenone, 2.5 µM oligomycin, or 100 nM antimycin A. (A) mitoSOX fluorescence. (B) Western blots of Cytochrome C release from mitochondria to cytosol, indicating induction of apoptosis. Antibodies in (b) and (c) were probed on the same blot, (a) on separate blot to avoid band overlap; (d) and (e) same blot, (f) on a separate blot to avoid band overlap. MW, molecular weight. Red boxes indicate regions corresponding to the protein of interest. (C) Cytochrome C densitometry. Results are presented as individual data points ± SEM of 8 (A) or 3-4 (C) per treatment group. *$P < 0.05$, ****$P < 0.001$ by one-way ANOVA and Tukey's posthoc test.

Supplemental Figure S6

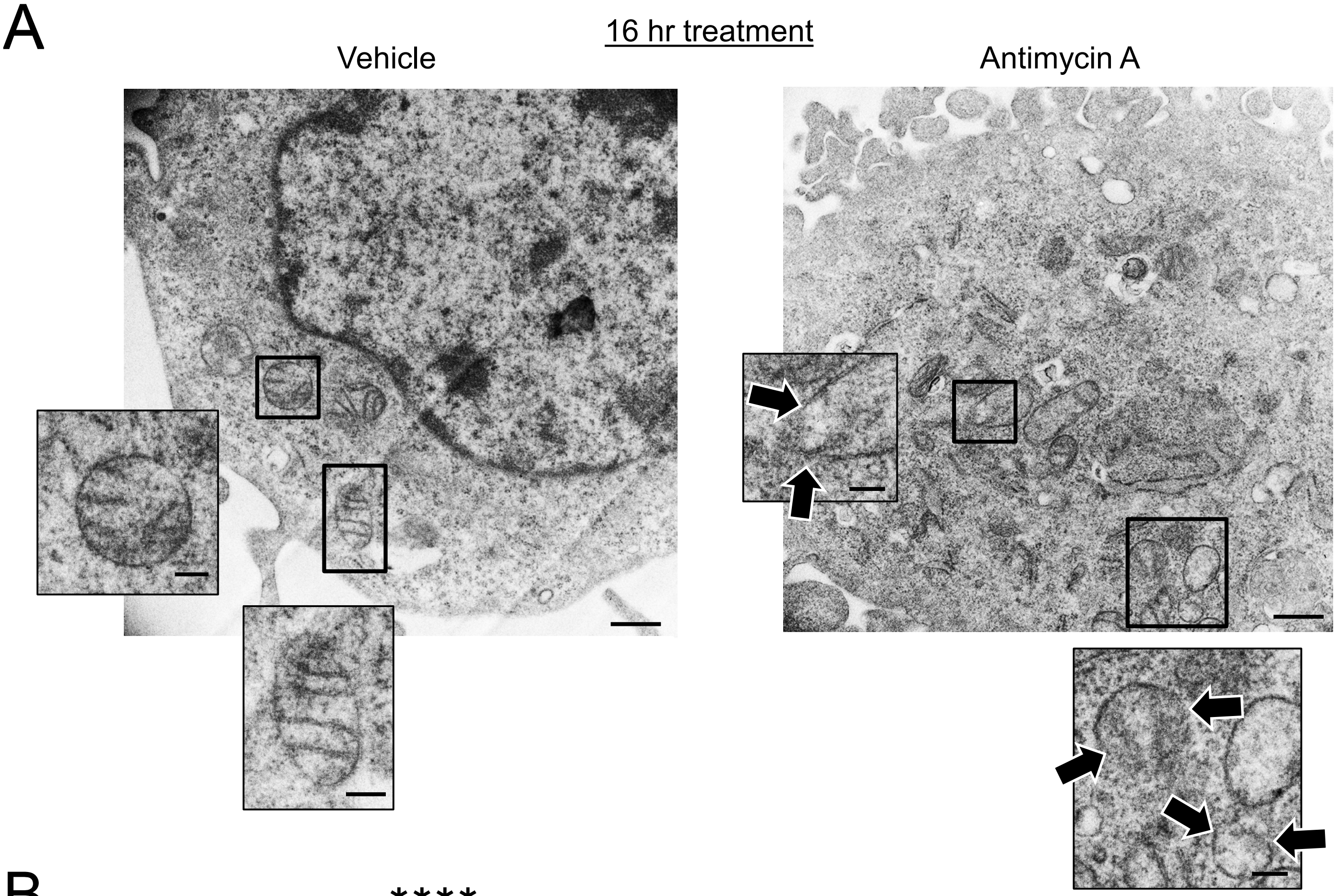

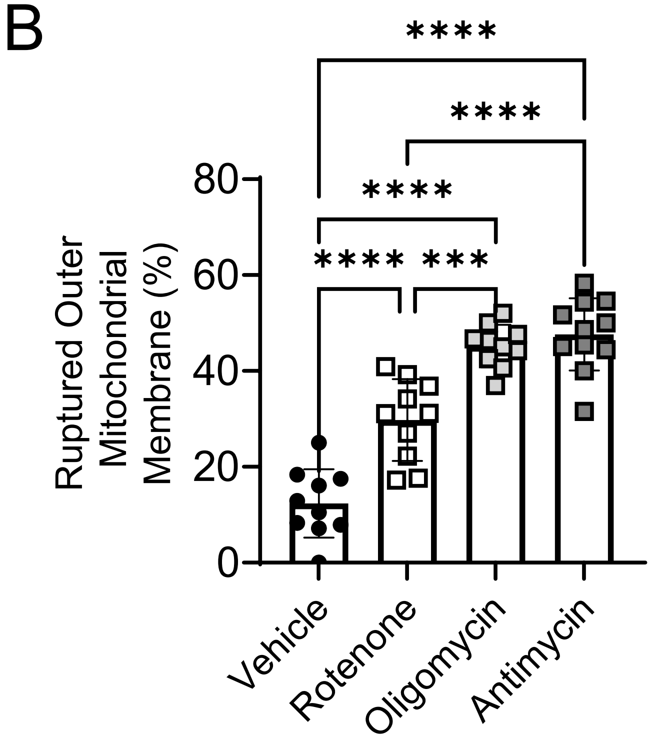

Figure S6. All mitochondrial drugs induce OMM rupture but oligomycin and antimycin A are most severe. Mode-K cells were treated with 500 nM rotenone, 2.5 µM oligomycin, or 100 nM antimycin A for 16 hr. (A) OMM rupture, as indicated by black arrows, was visualized by TEM imaging. Bar = 500 nm. (B) Quantification of OMM rupture by TEM imaging. Results are presented as individual data points ± SEM of 50 mitochondria/cell quantitated in 10 cells per treatment. ****$P < 0.001$ by one-way ANOVA and Tukey's posthoc test.

Supplemental Figure S7

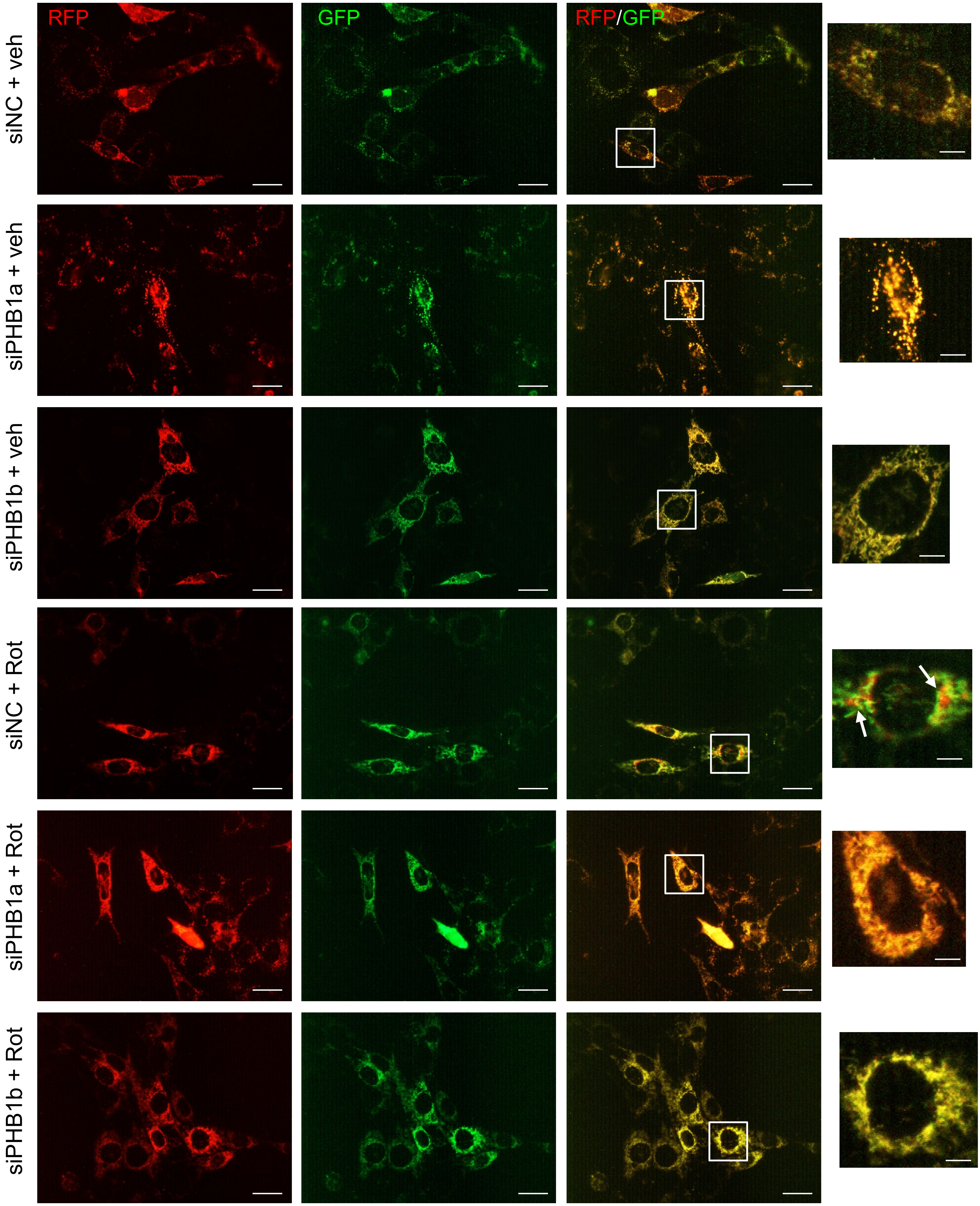

Figure S7. Additional images corresponding to Fig 3E. Mode-K cells were co-transfected with mito-RFP-EGFP during siPhb1 knockdown and treated with 500 nM rotenone for 2 hr to induce mitophagy. IF staining of mito-RFP-EGFP. Arrows: red signal. Scale bars: 50 µm, boxed pullout: 10 µm.

Supplemental Figure S8

Uncropped immunoblots in Fig. 3A

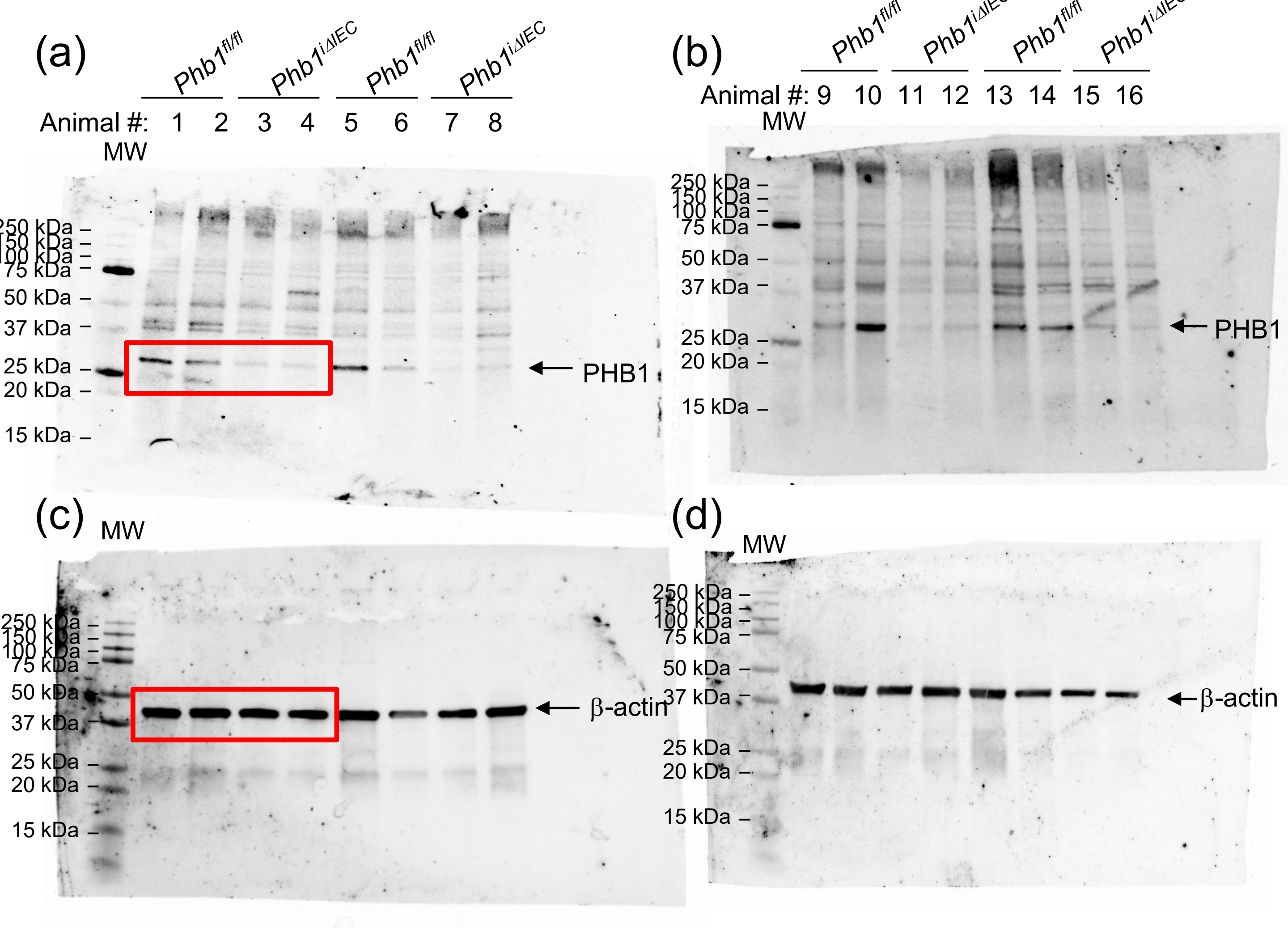

Figure S8. Original uncropped immunoblots. Immunoblots for (a, b) PHB1 and (c, d) β-actin in isolated intestinal epithelial cells from *Phb1*$^{fl/fl}$ or *Phb1*$^{i\Delta IEC}$ mice. Antibodies in (a) and (c), and (b) and (d) were probed on the same blot. Each lane represents IECs isolated from an individual mouse. MW, molecular weight. Red boxes indicate regions that were cropped and presented in figures within the manuscript.

Supplemental Figure S9

Uncropped immunoblots in Fig. 3A

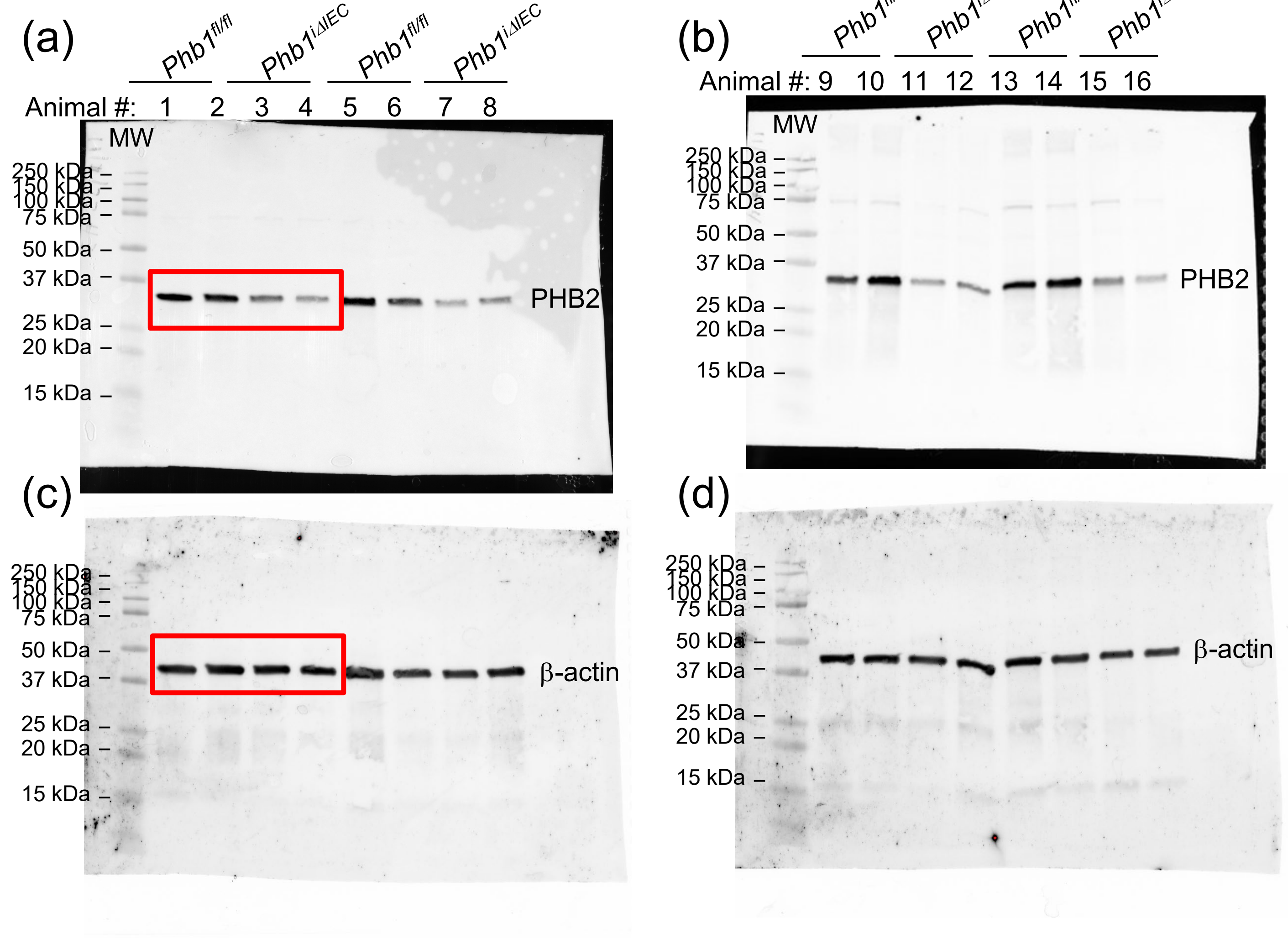

Figure S9. Original uncropped immunoblots. Immunoblots for (a, b) PHB2 and (c, d) β-actin in isolated intestinal epithelial cells from *Phb1*$^{fl/fl}$ or *Phb1*$^{i\Delta IEC}$ mice. Antibodies in (a) and (c), and (b) and (d) were probed on the same blot. Each lane represents IECs isolated from an individual mouse. MW, molecular weight. Red boxes indicate regions that were cropped and presented in figures within the manuscript.

Supplemental Figure S10

Uncropped immunoblots in Fig. 3B

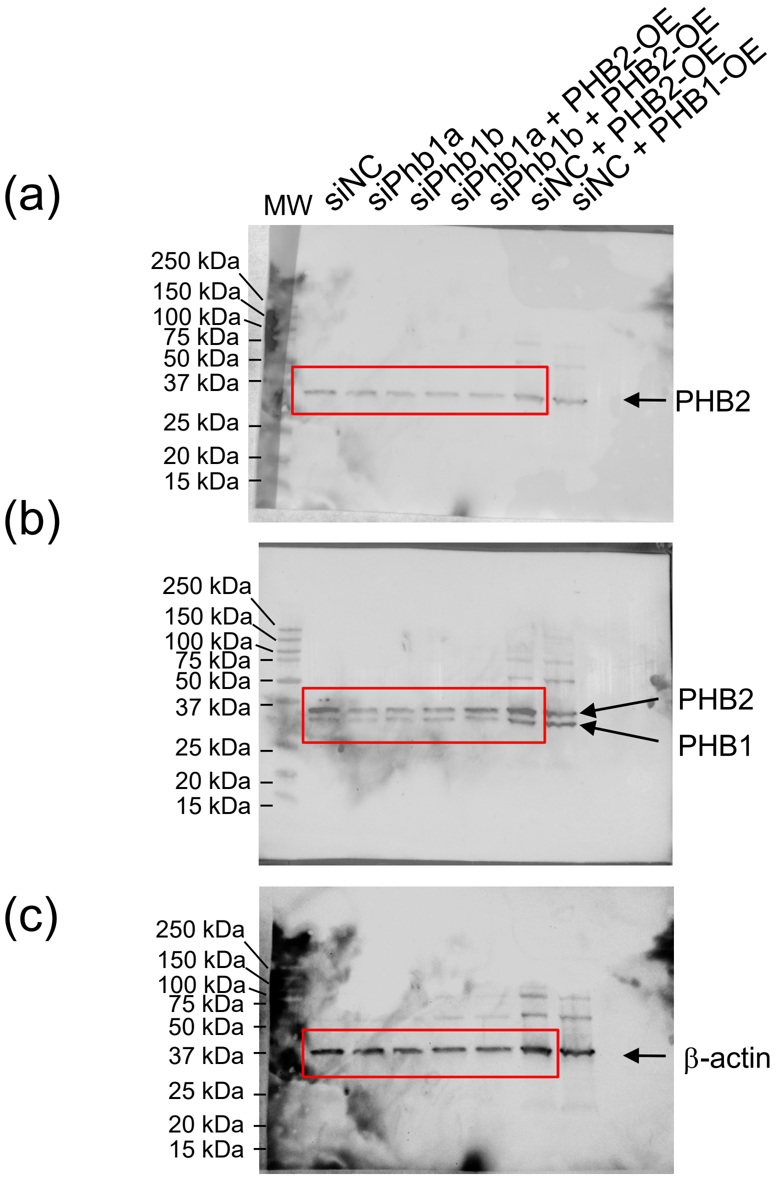

Figure S10. Original uncropped immunoblots. Immunoblots for (a) PHB2, (b) PHB1, and (c) β-actin in siPhb1 knockdown or siNC (control) Mode-K cells co-transfected with PHB2 or PHB1 overexpression vector (OE). Antibodies in (a), (b), and (c) were probed on the same blot that was stripped before probing for β-actin. MW, molecular weight. Red boxes indicate regions that were cropped and presented in figures within the manuscript.

Supplemental Figure S11

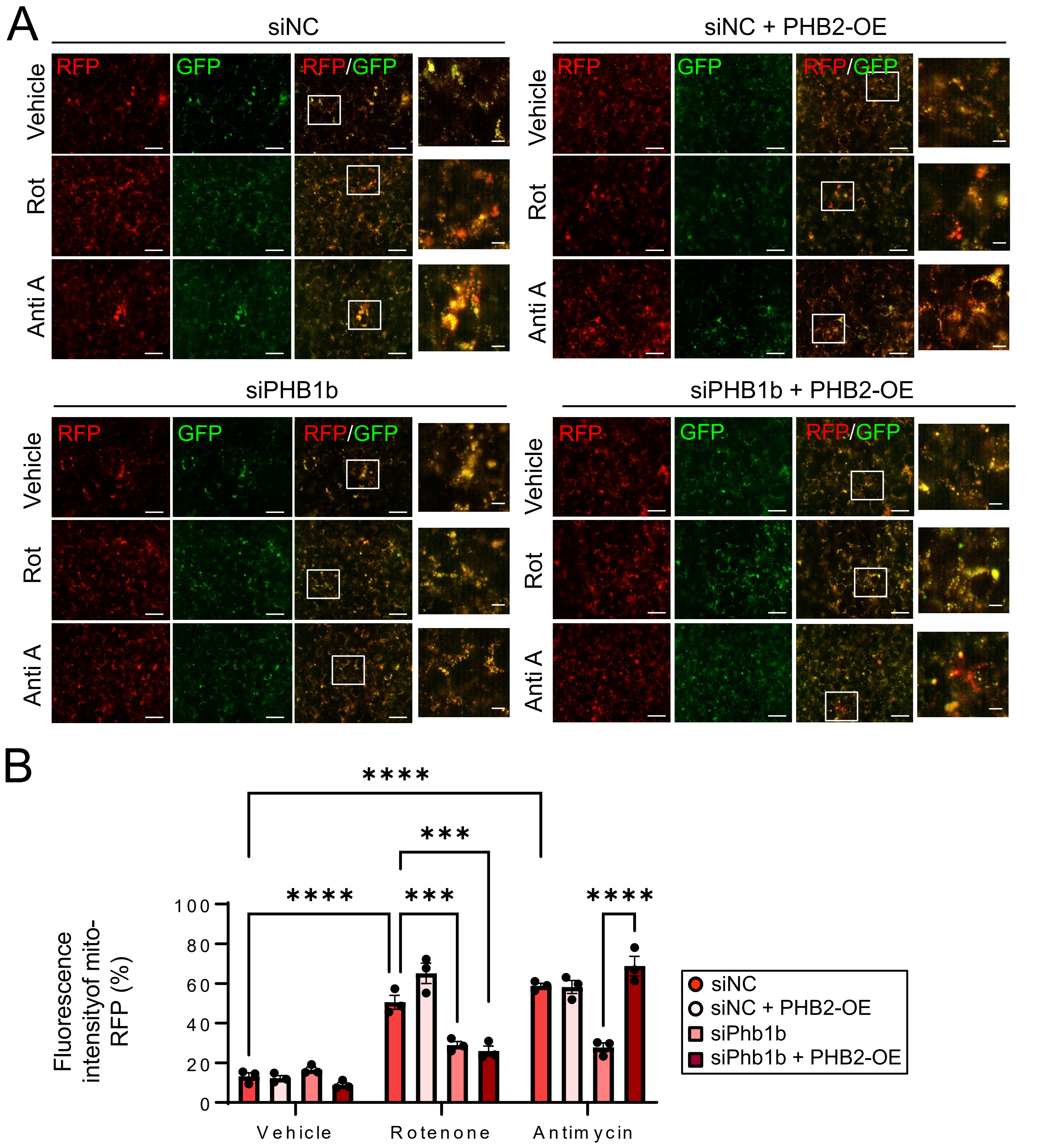

Figure S11. Mitophagy measured by mito-RFP-GFP expression. Mode-K cells were co-transfected with siPHB1b or siNC constructs, PHB2 overexpression plasmid (PHB2-OE) or empty vector control, and mito-RFP-EGFP and treated with 500 nM rotenone or 100 nM antimycin A for 2 hr to induce mitophagy. (A) IF staining of mito-RFP-EGFP. Scale bars: 100 μm, boxed pullout: 25 μm. (B) Quantitation of red pixel intensity using the average of 50 cells per well across 3 wells per treatment. Results are presented as individual data points ± SEM of 3 per treatment group. ***$P < 0.005$, ****$P < 0.001$ by one-way ANOVA and Tukey's posthoc test.

Supplemental Figure S12

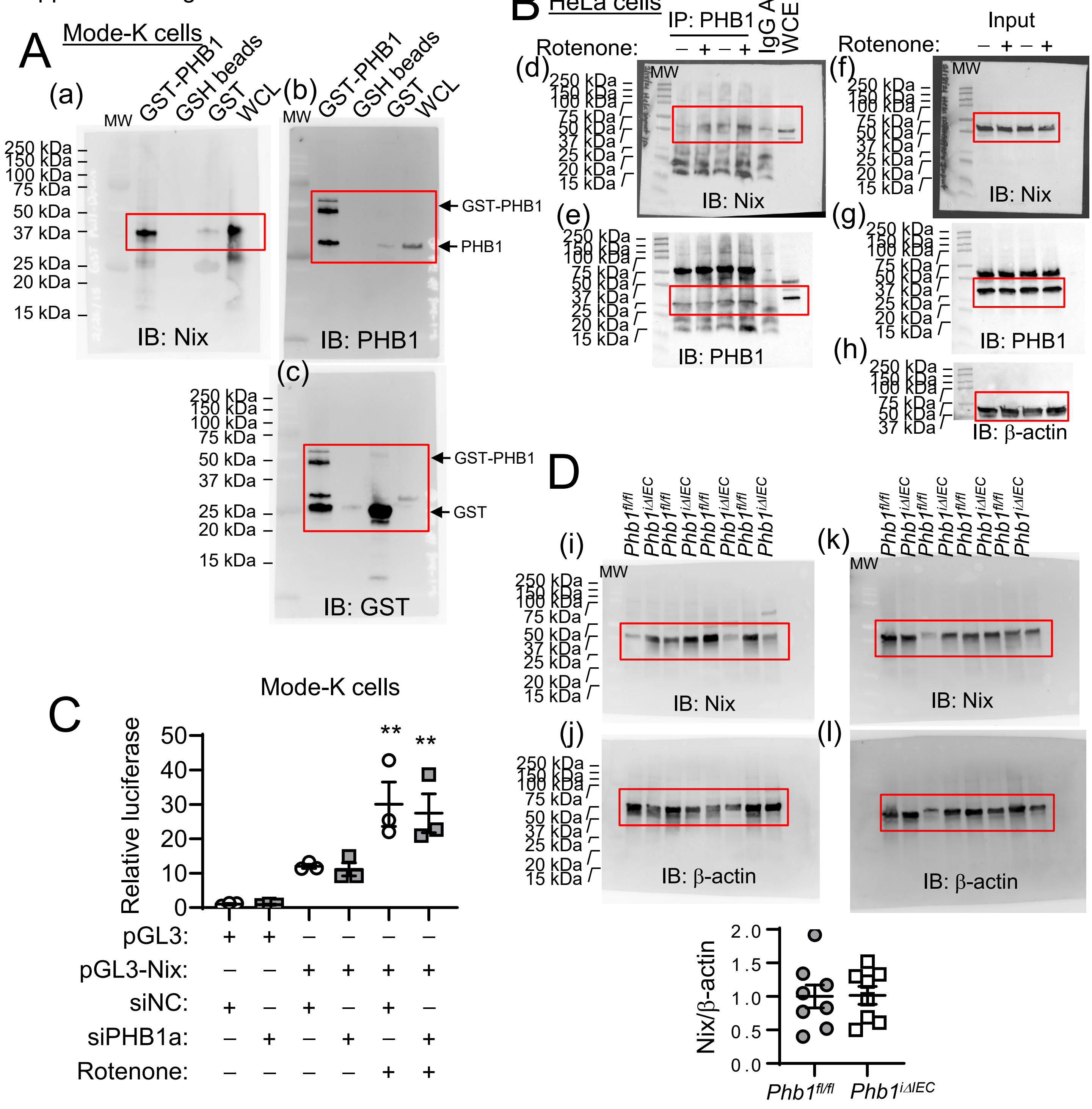

Figure S12. Nix interacts with PHB1. (A) Protein from isolated Mode-K cells was incubated with GST-PHB1 and analyzed by western blotting. (B) HeLa cells were treated with 500 nM rotenone for 2 hr. Protein was subjected to co-IP of PHB1 and Nix. (C) Mode-K cells were co-transfected with pGL3-Nix luciferase reporter and siPHB1a (Invitrogen) or siNC, treated with 500 nM rotenone for 2 hr, and assayed for relative firefly/renilla luciferase. (D) Nix expression in freshly isolated ileal IECs. Each lane represents one individual animal. Antibodies in (b) and (c) were probed on the same blot, (a) on a separate blot to avoid band overlap; (d) and (e) same blot, (f-h) same blot, (i) and (j) same blot, and (k) and (l) same blot. For (b), (d), (e), the image showing blot edges was not saved from an old imager. MW, molecular weight. Red boxes indicate regions corresponding to the protein of interest.

Supplemental Figure S13

Mode-K cells

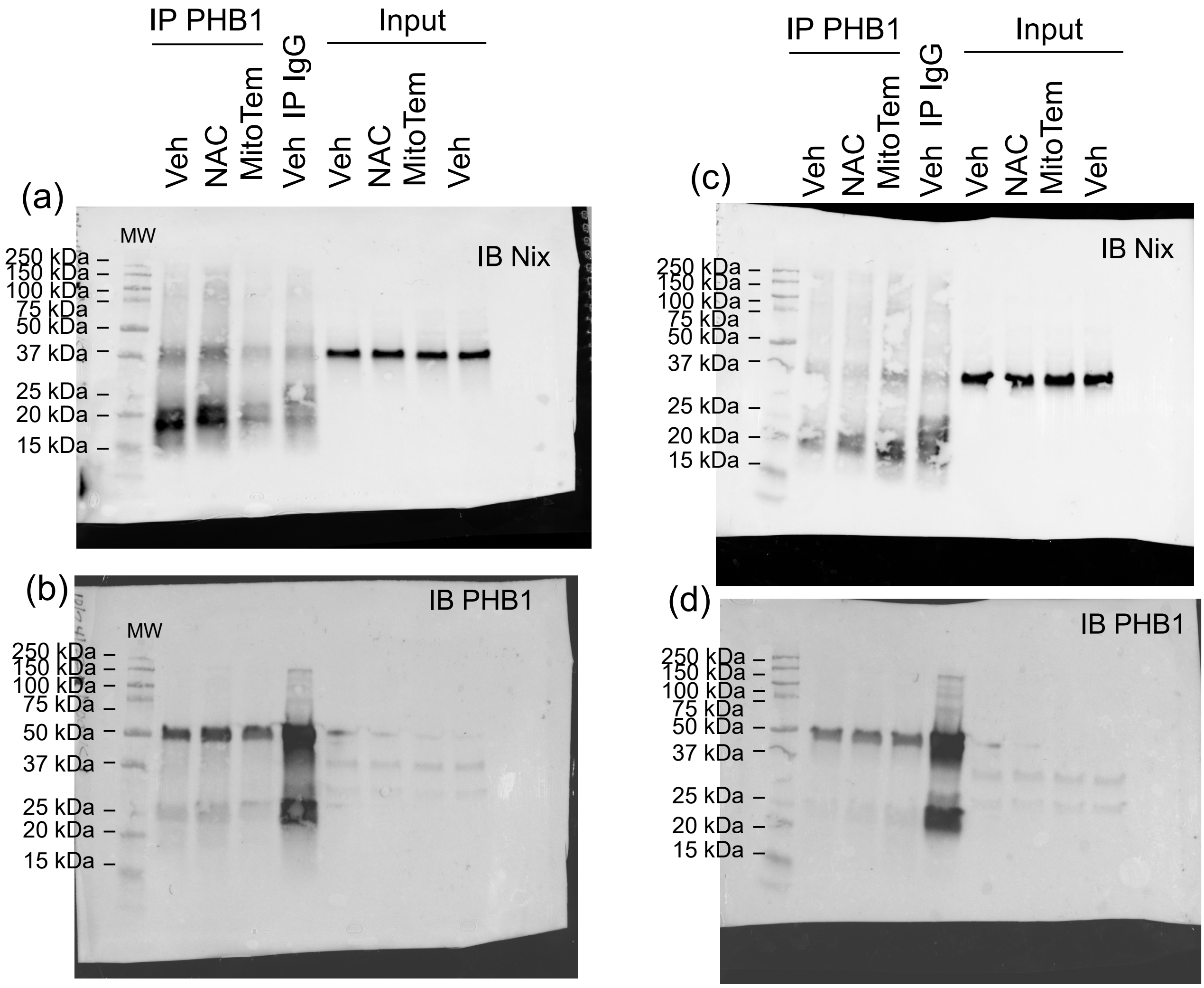

Figure S13. PHB1/Nix interaction during ROS scavenger treatment. Mode-K cells were treated with 10mM NAC or 25 nM mitoTempo for 2 hr. Protein was subjected to co-IP of PHB1 and Nix. Antibodies in (a) and (b) were probed on the same blot, (d) and (e) same blot. MW, molecular weight.

Supplemental Figure S14
Uncropped immunoblots in Fig. 4A

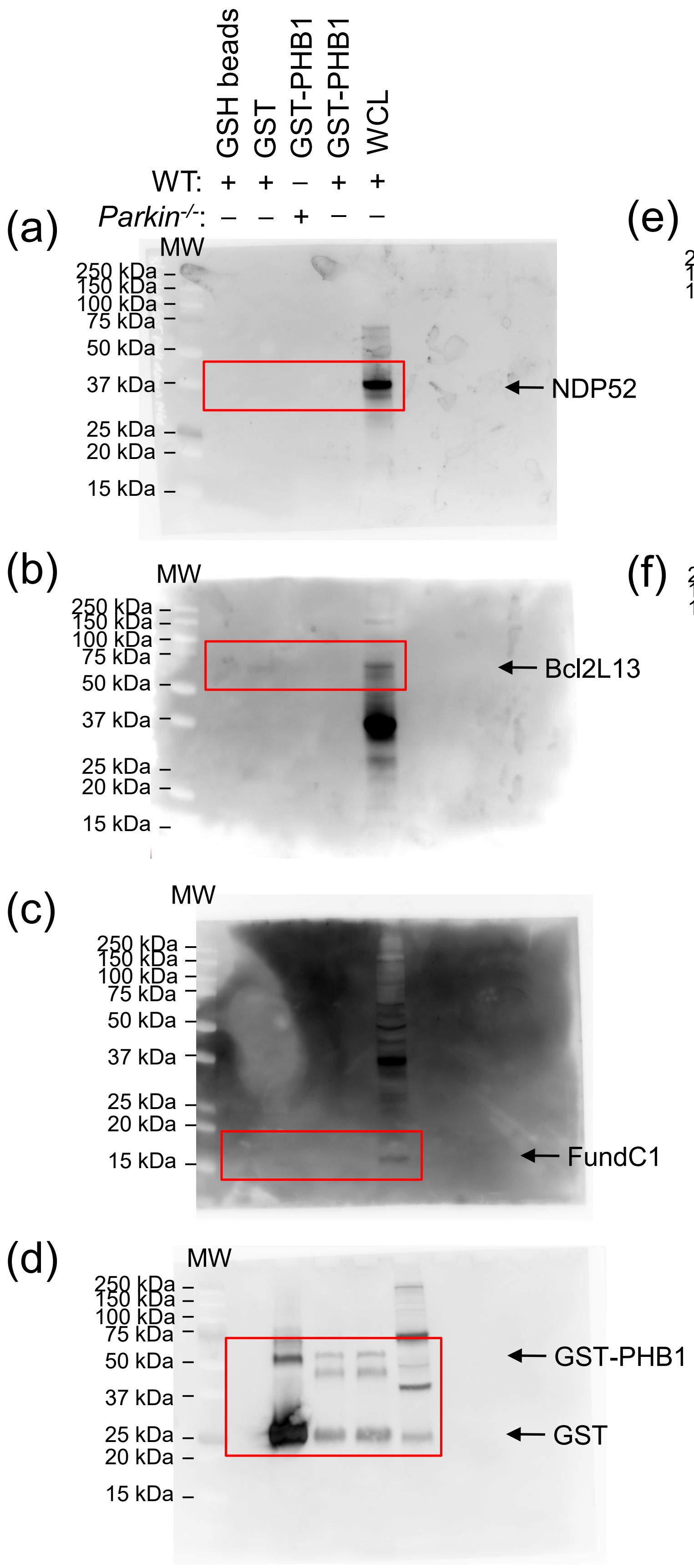

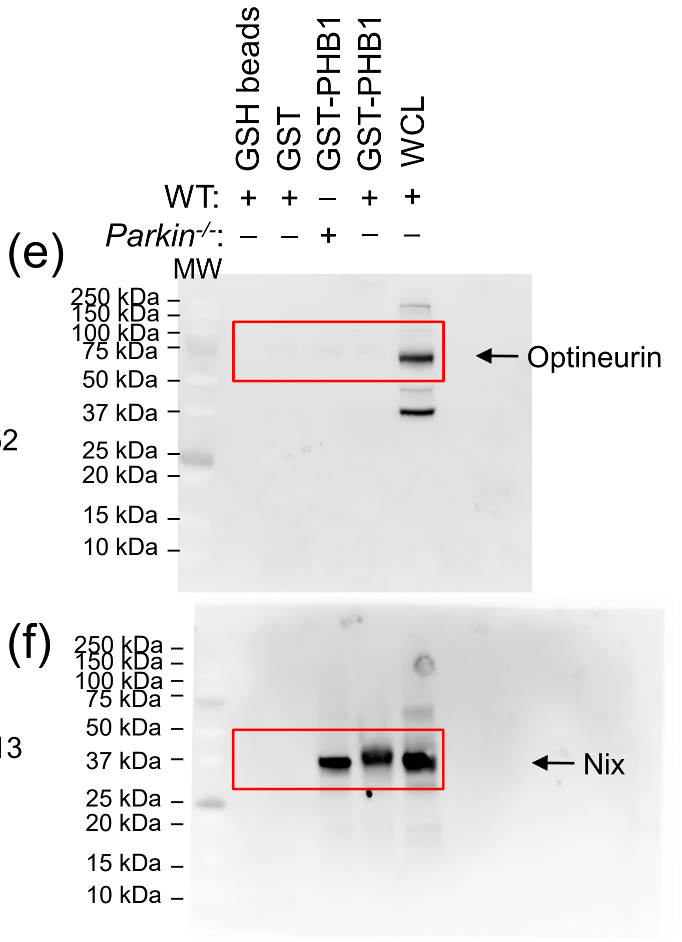

Figure S14. Original uncropped immunoblots. Immunoblots for (a) NDP52, (b) Bcl2L13, (c) FundC1, (d) GST, (e) Optineurin, and (f) Nix in GST pull downs using protein extracts from WT or *Parkin-/-* IECs. Antibodies in (a), (b), (c) and (d) were probed on the same blot. Optineurin (e) and Nix (f) were probed on a separate blot to prevent band overlap. For Optineurin, the image showing blot edges was not saved from an old imager. MW, molecular weight. Red boxes indicate regions that were cropped and presented in figures within the manuscript.

Supplemental Figure S15
Uncropped immunoblots in Fig. 4A input bands

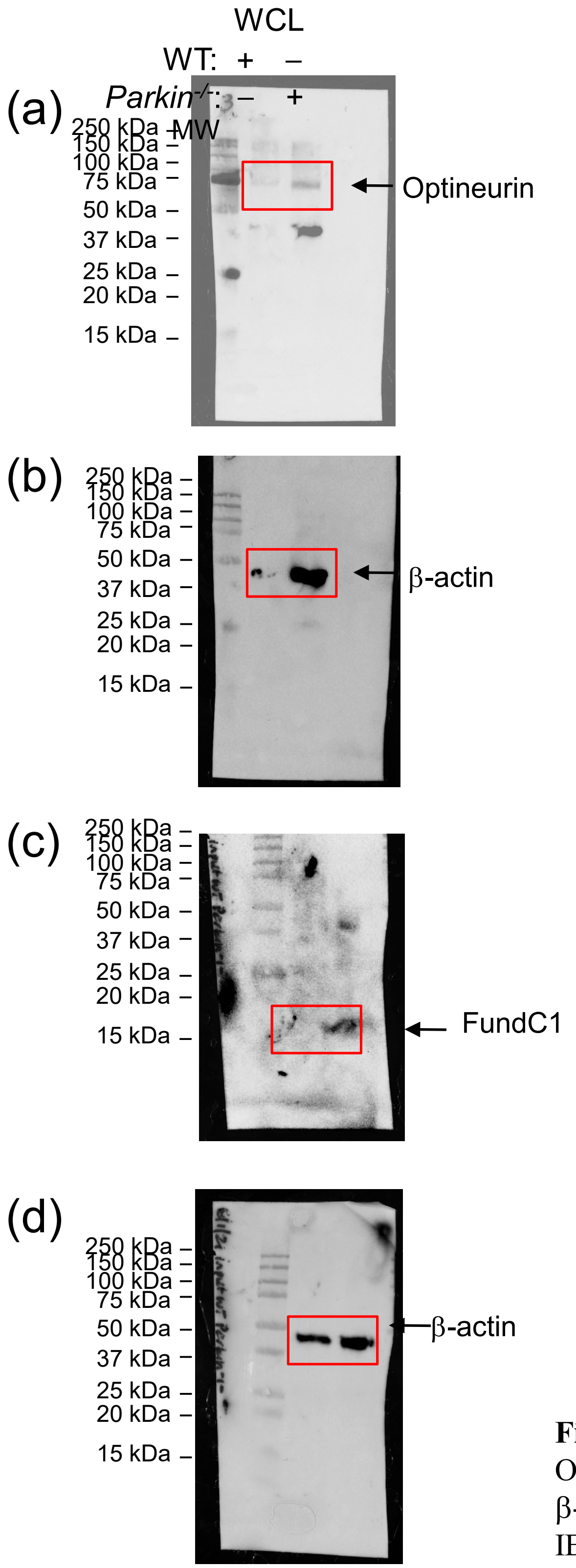

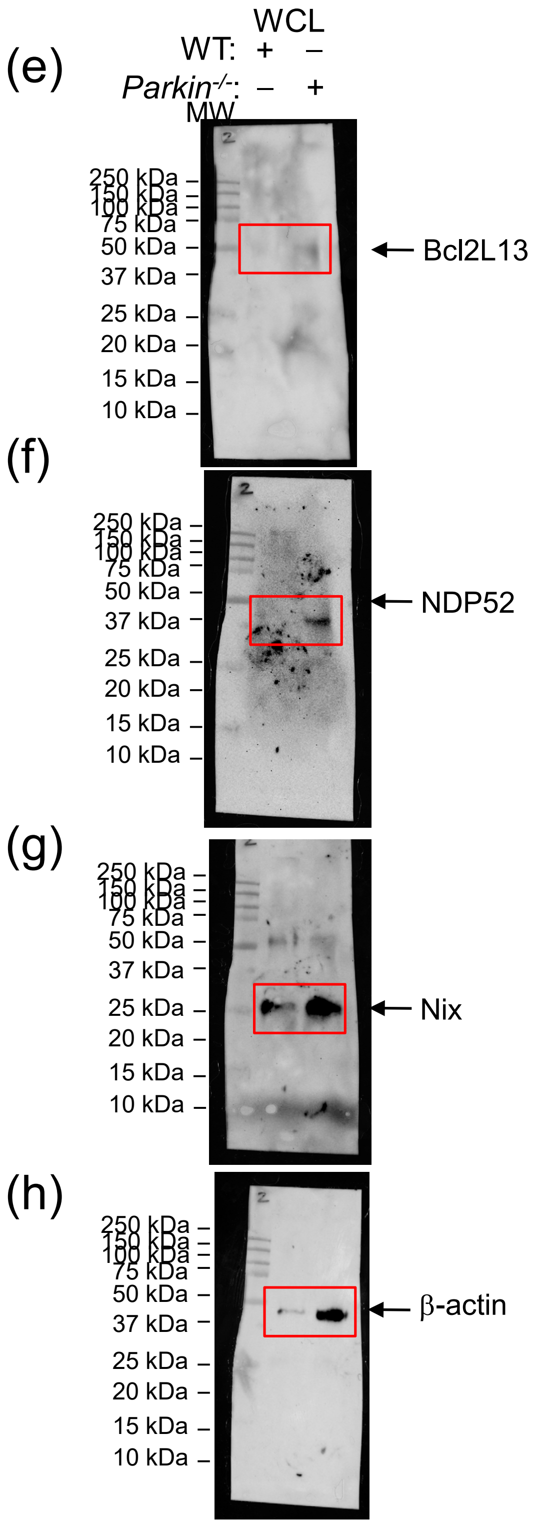

Figure S15. Original uncropped immunoblots for (a) Optineurin, (c) FundC1, (e) Bcl2L13, (f) NDP52, (g) Nix, and β-actin (b, d, h) in input protein extracts from WT or *Parkin-/-* IECs. Antibodies in (a) and (b) were probed on the same blot, (c) and (d) on the same blot, and (e), (f), (g), and (h) on the same blot to prevent band overlap. MW, molecular weight. Red boxes indicate regions that were cropped and presented in figures within the manuscript.

Supplemental Figure S16

Uncropped immunoblots in Fig. 4B

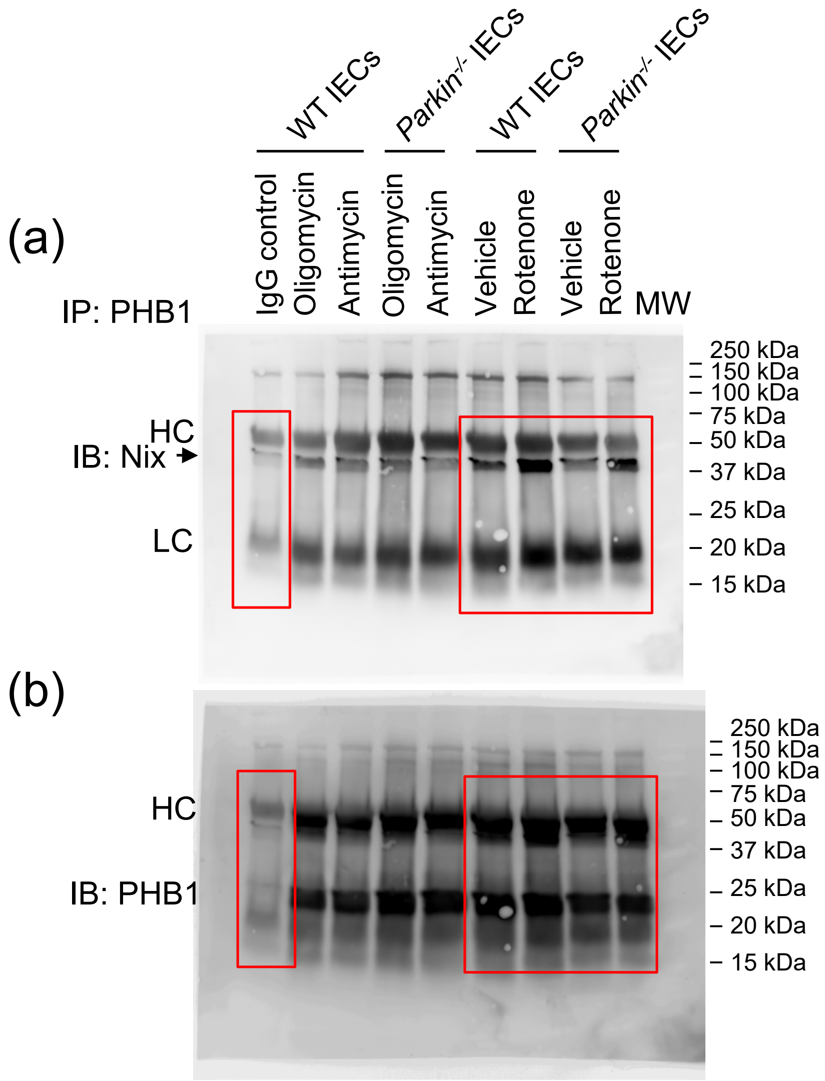

Figure S16. Original uncropped immunoblots for (a) Nix and (b) PHB1 in isolated intestinal epithelial cells treated with rotenone, antimycin, or oligomycin in short-term culture. Antibodies in (a) and (b) were probed on the same blot. HC: heavy chain; LC: light chain. MW, molecular weight. Red boxes indicate regions that were cropped and presented in figures within the manuscript.

Supplemental Figure S17

Uncropped immunoblots in Fig. 4B - input

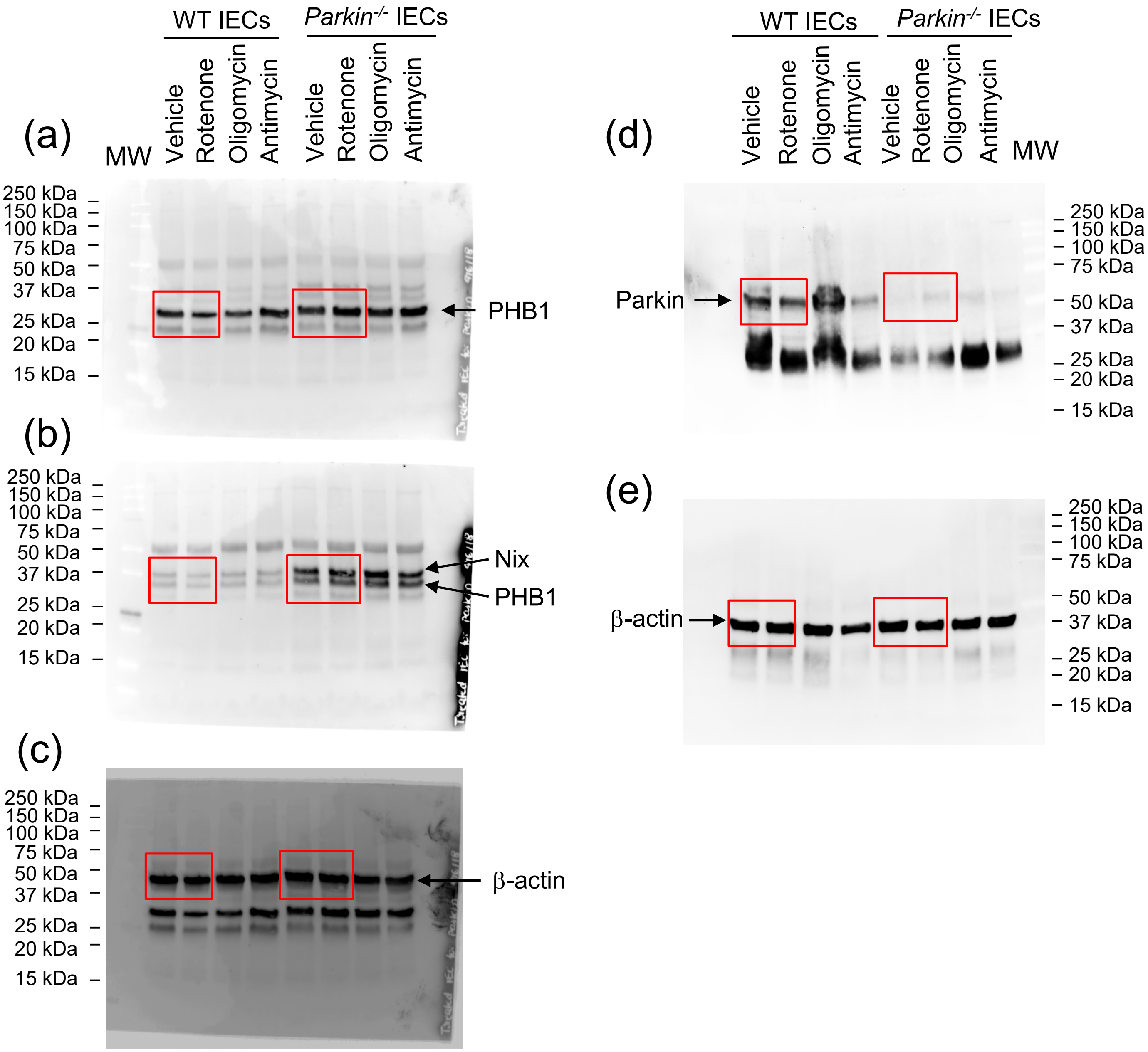

Figure S17. Original uncropped immunoblots for (a) PHB1, (b) Nix, (d) Parkin, and (c, e) β-actin in isolated intestinal epithelial cells from WT or *Parkin*$^{-/-}$ mice treated with rotenone, antimycin, or oligomycin in short-term culture. Antibodies in (a), (b), and (c) were probed on the same blot. Antibodies in (d) and (e) were probed on a separate blot to prevent band overlap. Images showing blot edges for (d) and (e) were not saved from an old imager. MW, molecular weight. Red boxes indicate regions that were cropped and presented in figures within the manuscript.

Supplemental Figure S18

Uncropped immunoblots in Fig. 4C

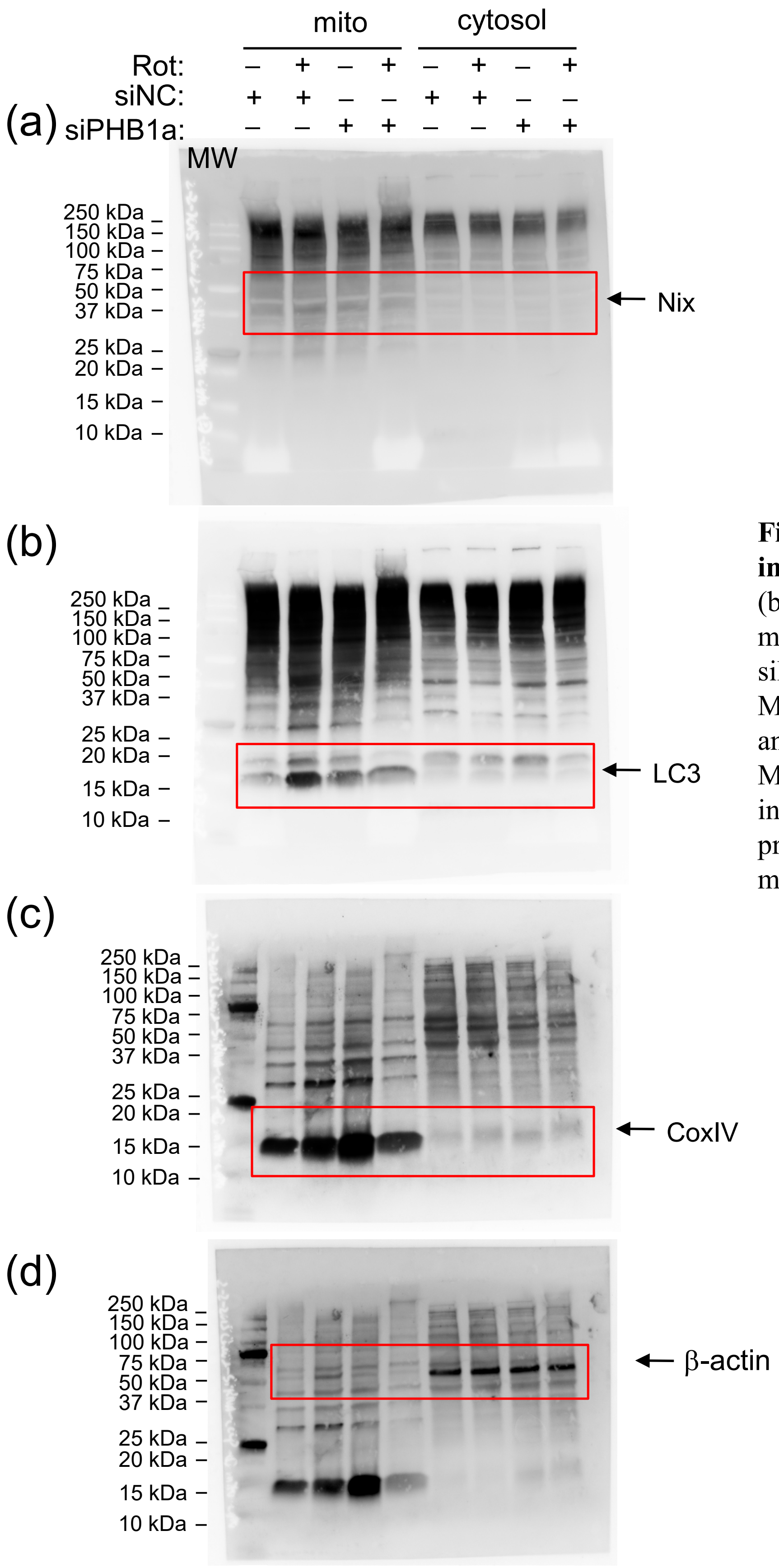

Figure S18. Original uncropped immunoblots. Immunoblots for (a) Nix, (b) LC3, (c) CoxIV, and (d) β-actin in mitochondrial and cytosolic extracts from siPhb1 knockdown or siNC (control) Mode-K cells treated with rotenone. All antibodies were probed on the same blot. MW, molecular weight. Red boxes indicate regions that were cropped and presented in figures within the manuscript.

Supplemental Figure S19

Blots in Figure 5B

Figure S19. Full immunoblots for (a, c) Nix and (b, d) GFP in Mode-K cells expressing GFP-PHB1 deletion constructs, immunoprecipitated for GFP and blotted for Nix and GFP. (a) and (b) were probed on the same blot. (c) and (d) were proved on a separate blot. MW, molecular weight. Red boxes indicate regions that were cropped and presented in figures within the manuscript. Images showing blot edges were not saved from an old imager.

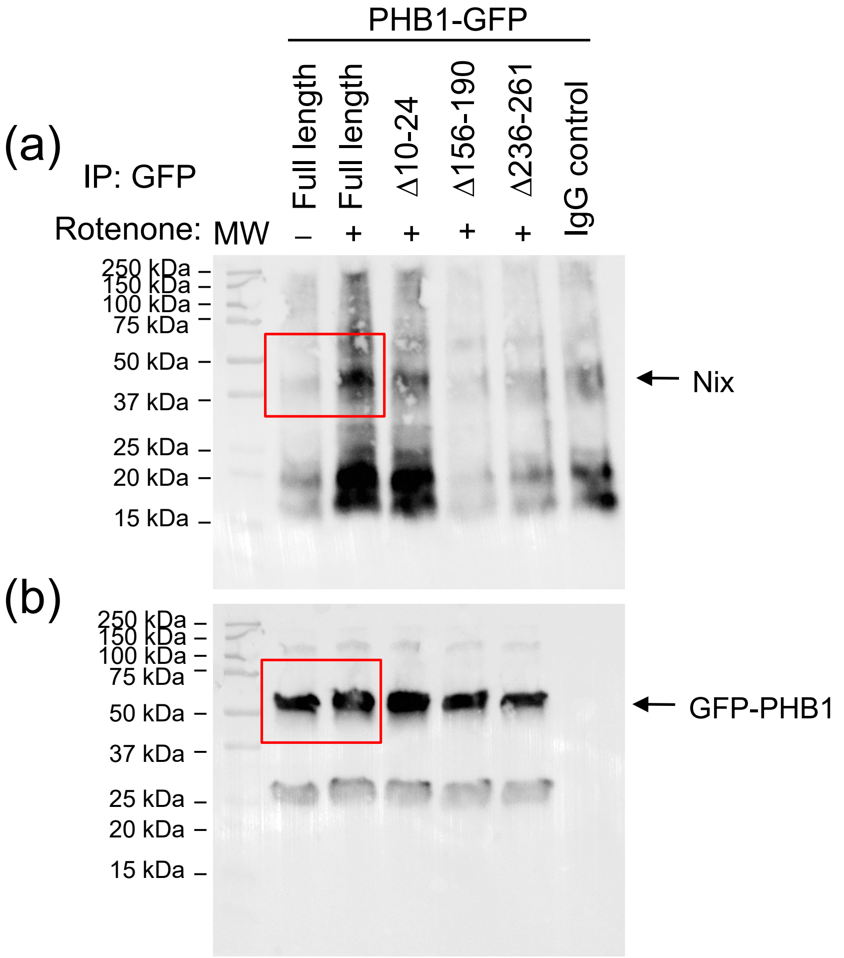

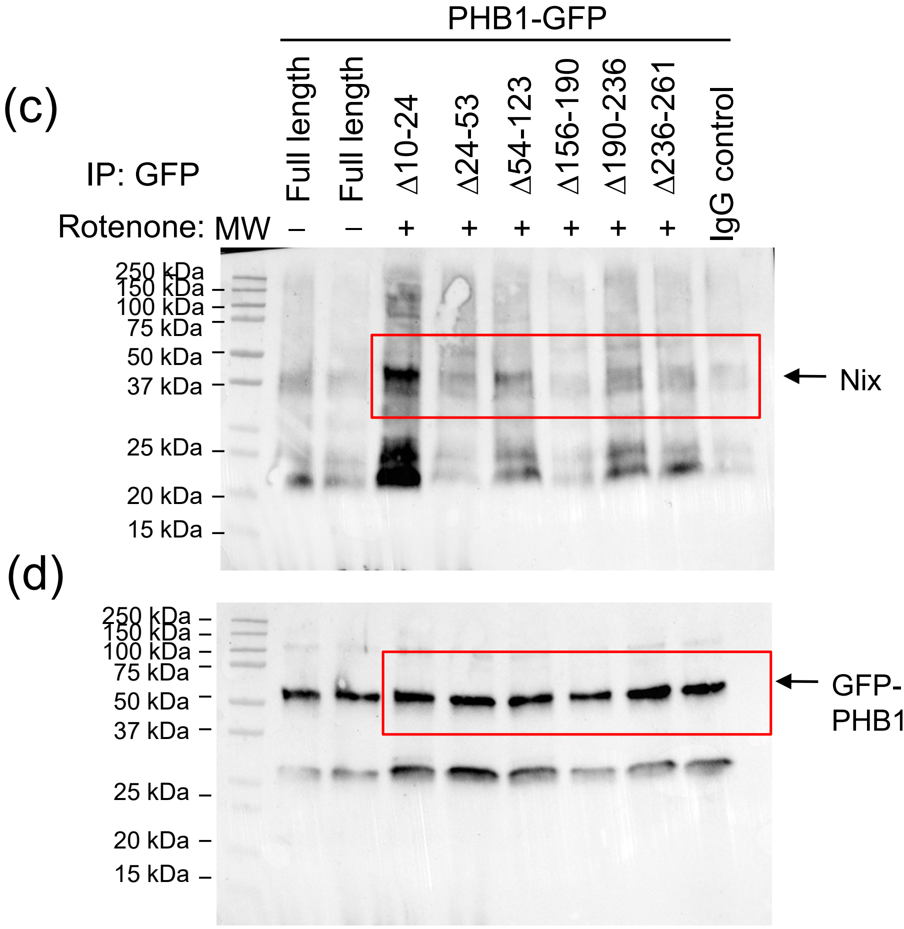

Supplemental Figure S20

Uncropped immunoblots in Fig. 5B input blots

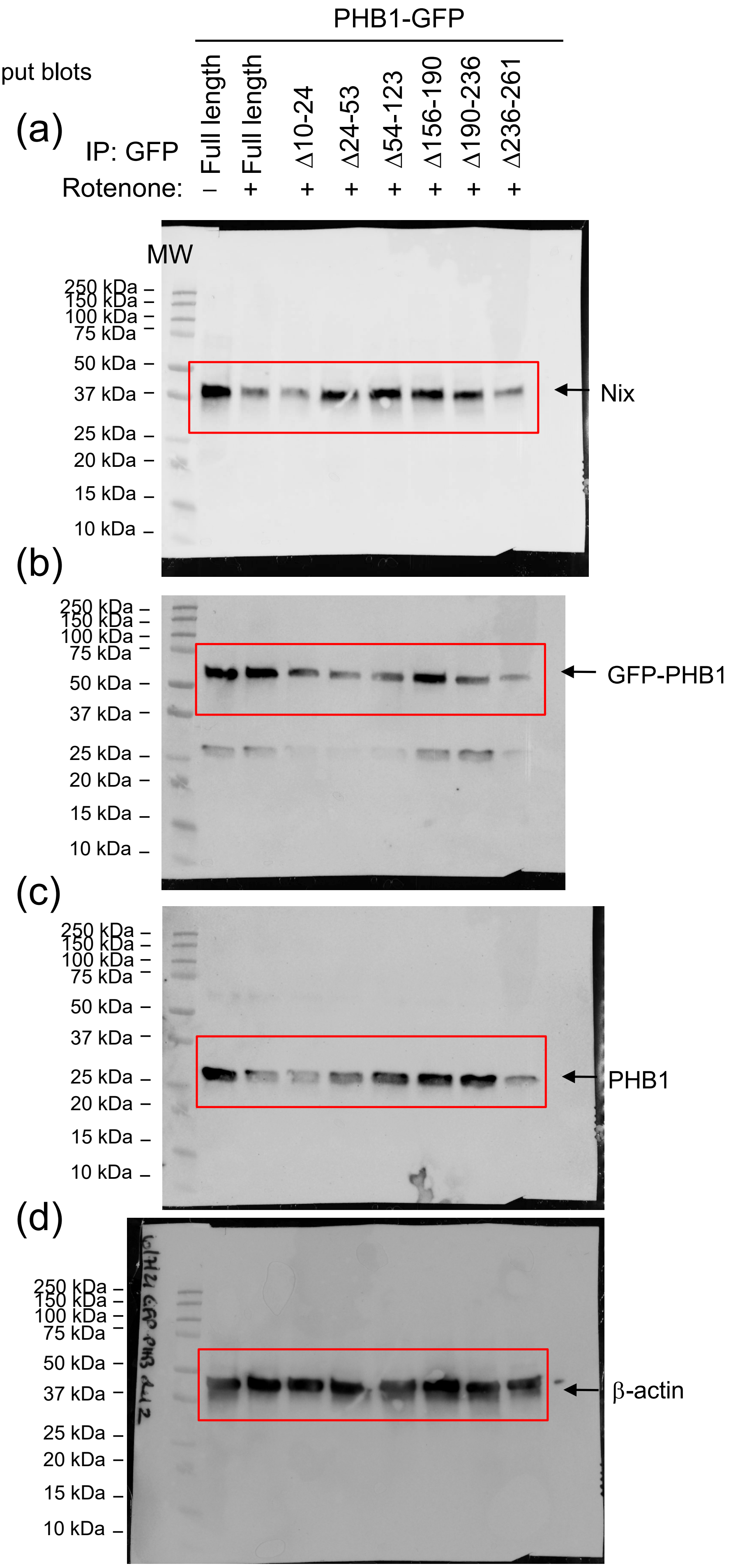

Figure S20. Original uncropped immunoblots. Representative immunoblots for (a) Nix, (b) GFP, (c) PHB1, and (d) β-actin in Mode-K cells expressing GFP-PHB1 deletion constructs and treated with rotenone. All antibodies were probed on the same blot. MW, molecular weight. Red boxes indicate regions that were cropped and presented in figures within the manuscript.

Supplemental Figure S21

Uncropped immunoblots in Fig. 5D

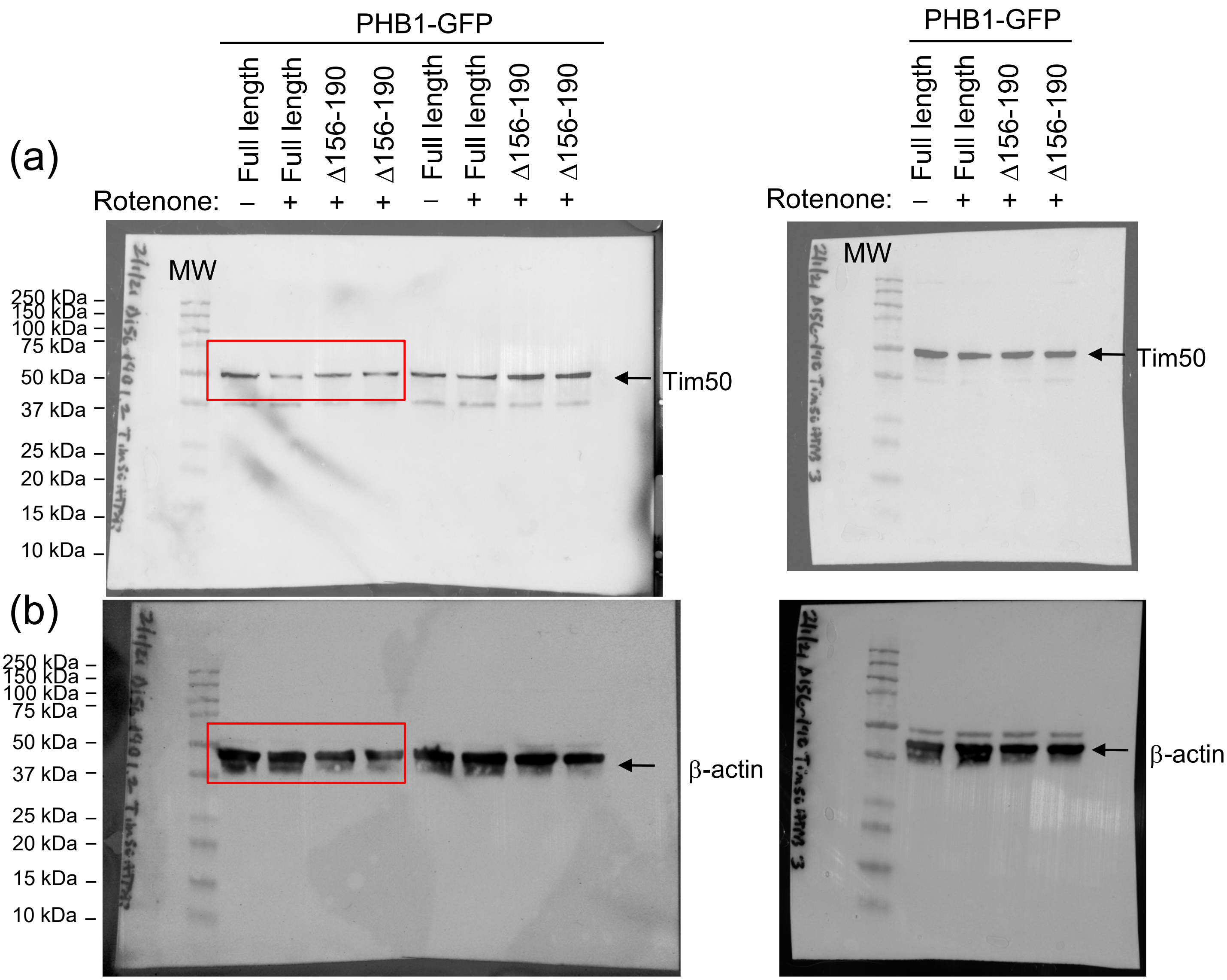

Figure S21. Original uncropped immunoblots. Representative immunoblots for (a) Tim50 and (b) β-actin in Mode-K cells expressing GFP-PHB1 full length or 156-190 deletion constructs and treated with rotenone. All antibodies were probed on the same blot. MW, molecular weight. Red boxes indicate regions that were cropped and presented in figures within the manuscript.

Supplemental Figure S22

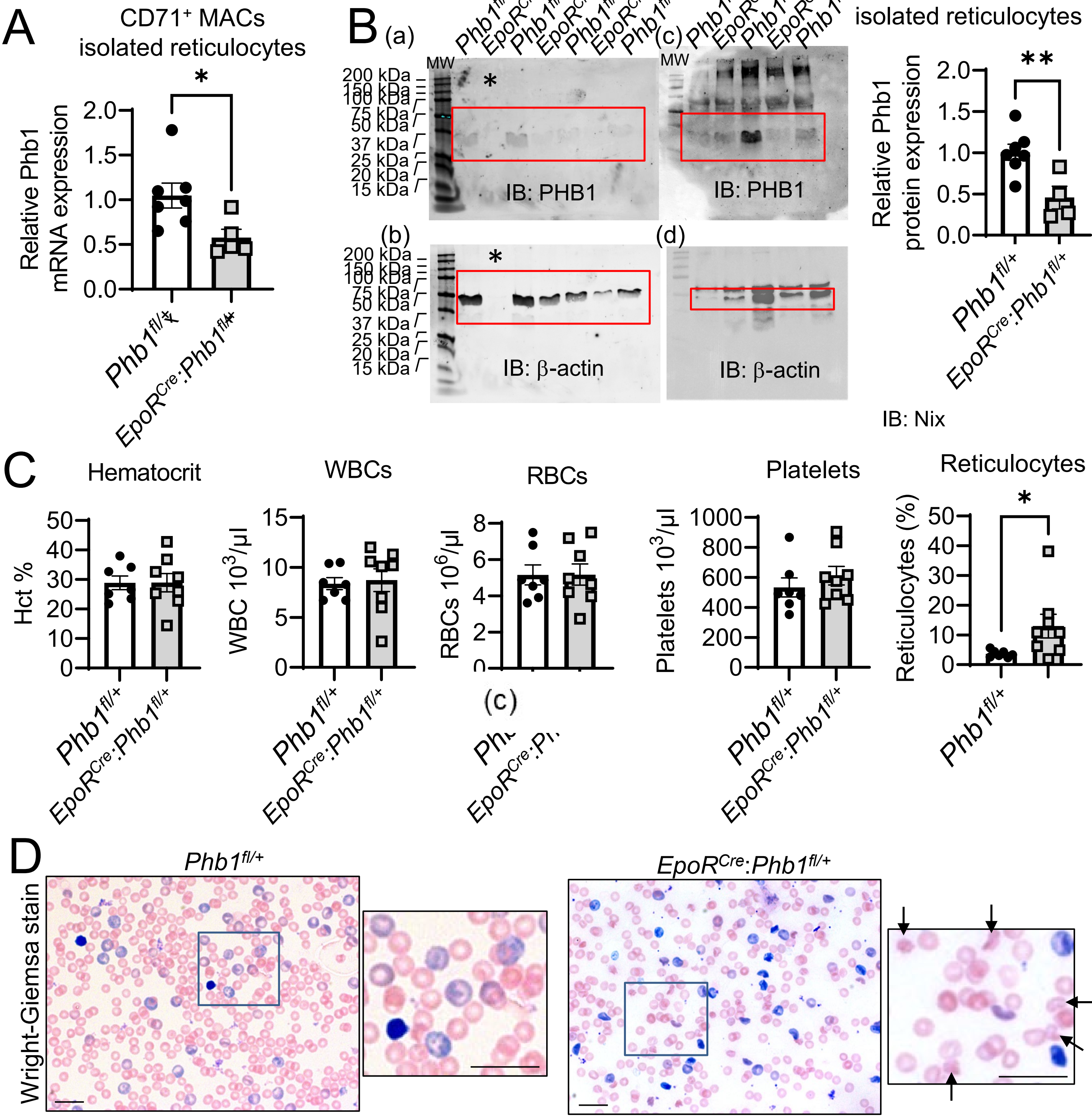

Figure S22. Characterization of Phb1 expression in *EpoR*^{Cre}*:Phb1*$^{fl/+}$ reticulocytes. Phb1 mRNA (A) or protein (B) expression in CD71^{+} MACS magnetic bead column-isolated reticulocytes. Antibodies in (a) and (b), (c) and (d) were probed on the same blot. Image showing blot edges was not saved from an old imager. MW, molecular weight. Red boxes indicate regions corresponding to the protein of interest. *denotes sample not included in densitometry since no β-actin was present. Each lane represents reticulocytes isolated from an individual mouse. (C) Complete blood count analysis. (D) Fresh blood smears stained with Wright-Giemsa. Arrows denote abnormally shaped erythrocytes. Scale bars: 20 µm. Results are presented as individual data points ± SEM of 7 *Phb1*$^{fl/+}$ or 4-5 *EpoR*^{Cre}*:Phb1*$^{fl/+}$ mice (A, B) or 7 *Phb1*$^{fl/+}$mice or 8 *Phb1*$^{fl/+}$*:EpoR*^{Cre} mice per group (C). *$P < 0.05$, **$P < 0.01$ by unpaired 2-tailed Student's *t* test.

Supplemental Figure S23

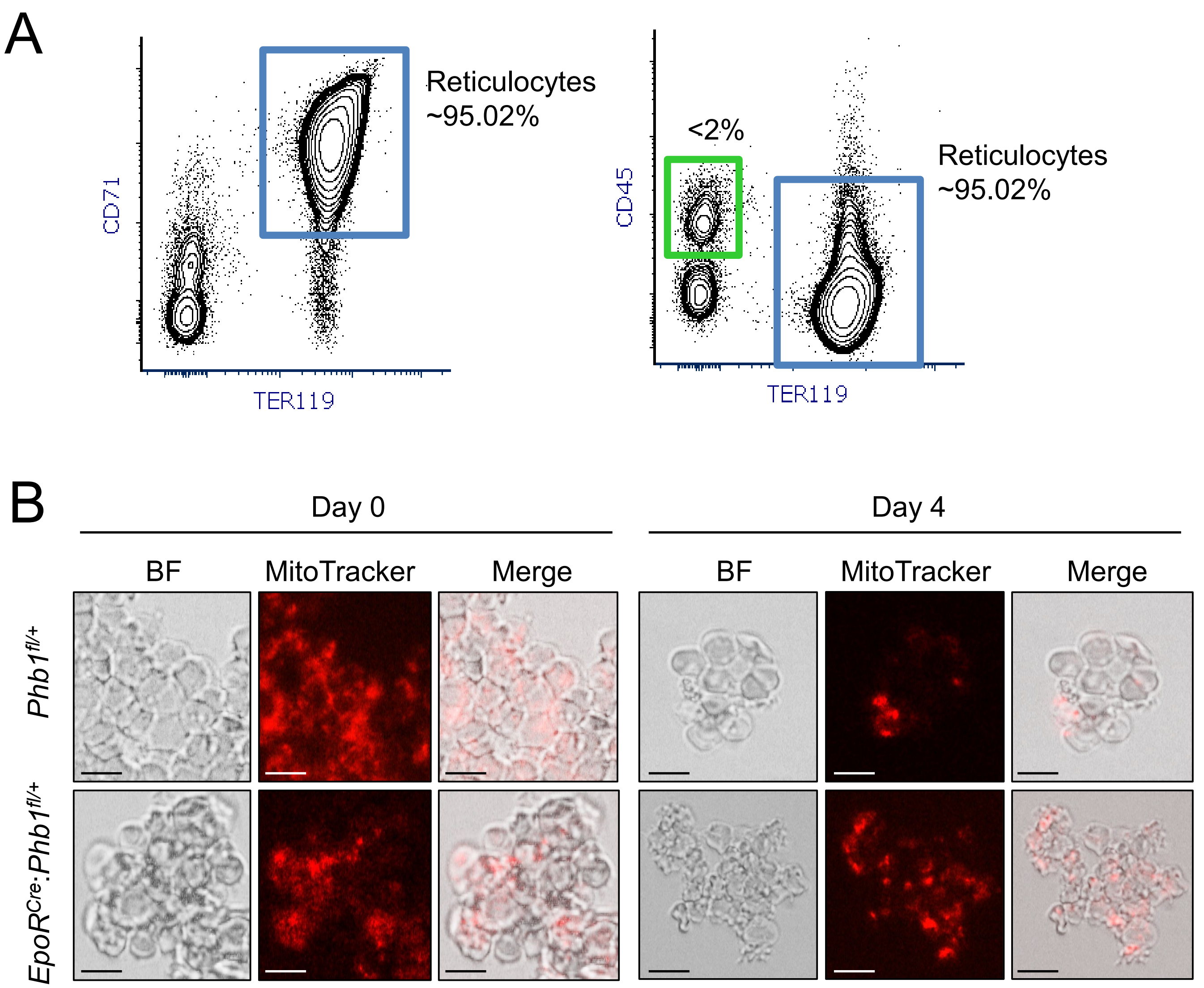

Figure S23. PHB1 mediates Nix-induced mitochondrial clearance during reticulocyte maturation. (A) Representative flow cytometry plots of reticulocytes purified by $CD71^+$ MACS magnetic bead/column isolation. Plots are gated on $7AAD^-$ cells. TER119 marks cells of the erythroid lineage, CD71 marks reticulocytes which constitutes 95% of the isolated cells, and $CD45^+$ indicates low contamination (<2%) by immune cells. (B) Immunofluorescence imaging of reticulocytes cultured for 4 days and stained with MitoTracker Red. BF, bright field. Scale bars: 10 µm.
